# Phosphate‐Engineered DNA Reporters Boost CRISPR‐Cas12a *Trans*‐Cleavage for Rapid and Sensitive Nucleic Acid Detection

**DOI:** 10.1002/advs.78445

**Published:** 2026-10-25

**Authors:** Rui Yang, Chong Guo, Minjie Pei, Jiongyu Zhang, Xin Guan, Shuo Zhang, Xingye Liu, Kurt T. Schalper, Lori Avery, Rocco LaSala, David Banach, Changchun Liu

**Affiliations:** ^1^ Department of Biomedical Engineering University of Connecticut Health Center Farmington Connecticut USA; ^2^ Department of Biomedical Engineering University of Connecticut Storrs Connecticut USA; ^3^ Department of Pathology and Laboratory Medicine University of Connecticut Health Center Farmington Connecticut USA; ^4^ Department of Medicine Division of Infectious Diseases University of Connecticut Health Center Farmington Connecticut USA

**Keywords:** chemistry, crispr, dna, fluorescence, fluorescence intensity, molecular diagnostics, nucleic acid

## Abstract

CRISPR–Cas12a molecular diagnostics rely on target‐activated collateral cleavage of single‐stranded DNA (ssDNA) reporters to generate fluorescence signals, yet the mechanisms governing reporter substrate processing remain poorly understood. Here we show that Cas12a *trans*‐cleavage involves both endonucleolytic cleavage and exonucleolytic trimming. We further demonstrate that terminal phosphate modification of ssDNA substrates accelerates substrate cleavage activity and modulates *trans*‐cleavage kinetics, thereby enhancing fluorescence output. Guided by this mechanistic insight, we engineered fluorophore–quencher ssDNA reporters bearing terminal phosphate modifications and identified a 3′‐phosphate‐engineered reporter that enables faster signal generation and higher fluorescence intensity. Incorporation of the engineered reporter into the CRISPR–Cas12a system enabled the development of Phos‐CRISPR (**Phos**phate‐engineered DNA reporters boost **CRISPR**), a rapid, sensitive, and specific nucleic acid detection platform that achieves results within 20 min, approximately threefold faster than conventional CRISPR assays. Applied to herpes simplex virus 1 detection, the Phos‐CRISPR combined with recombinase polymerase amplification achieved a limit of detection of 20 aM and accurately identified clinical swab samples. These findings provide a general framework for understanding Cas12a *trans*‐cleavage activity and establish a mechanistically informed strategy for enhancing next‐generation CRISPR diagnostics.

## Graphical abstract

We uncover dual CRISPR‐Cas12a *trans*‐cleavage activities: endonucleolytic cleavage and 3′→5′ exonucleolytic trimming of ssDNA reporters. Mechanism‐guided 3′‐phosphate engineering accelerates substrate cleavage while favoring endonucleolytic processing, enabling Phos‐CRISPR, a rapid (20 min), highly sensitive, and specific platform for nucleic acid detection.

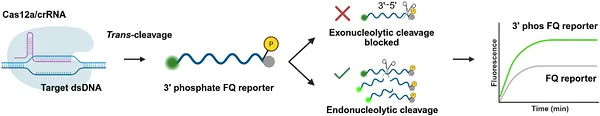

## 1 Introduction

CRISPR–Cas12a has developed into a promising platform for nucleic acid detection owing to its programmable DNA recognition and target‐activated collateral nuclease activity [1, 2, 3]. Cas12a is a class 2 type V CRISPR‐associated nuclease that uses a single CRISPR RNA (crRNA) to recognize complementary nucleic acid targets, such as double‐stranded DNA (dsDNA), adjacent to a protospacer‐adjacent motif (PAM) [4, 5]. Upon recognition of a specific nucleic acid target, the Cas12a–crRNA complex becomes activated, executes *cis*‐cleavage of the target nucleic acid, and subsequently triggers indiscriminate *trans*‐cleavage of single‐stranded DNA (ssDNA) substrates [6, 7]. This collateral activity forms the biochemical basis of a broad class of CRISPR‐based diagnostic technologies in which cleavage of fluorophore–quencher (FQ)‐labeled ssDNA reporters generates fluorescence signals for sensitive and specific nucleic acid detection [8, 9, 10].

Early CRISPR‐Cas12a assay platforms, including DETECTR [6] and HOLMES [9], coupled Cas12a *trans*‐cleavage with isothermal amplification strategies to achieve sensitive and specific nucleic acid detection [11, 12]. These studies established Cas12a as a versatile molecular detection module and stimulated the development of Cas12a‐based assays for the detection of pathogens, cancer biomarkers, genetic variants, and other clinically relevant nucleic acid targets at the point of care [13, 14, 15]. Subsequent studies further improved assay performance through optimization of amplification methods, reaction conditions, and reporter architectures [16, 17, 18]. Because the ssDNA reporter serves as the immediate substrate for activated Cas12a, its molecular architecture is expected to directly govern signal generation [19, 20, 21]. In most current CRISPR‐Cas12a systems, researchers evaluate *trans*‐cleavage activity using bulk fluorescent ssDNA reporters labeled with fluorophore–quencher pairs, making reporter design a central determinant of assay sensitivity and kinetics [22, 23].

Despite widespread application, Cas12a *trans*‐cleavage has long been considered indiscriminate toward ssDNA substrates [6, 7]. In this model, following target‐dependent activation, Cas12a is assumed to degrade ssDNA reporters without sequence or structural preference. However, the detailed cleavage patterns and substrate processing behaviors underlying this activity remain insufficiently characterized. In particular, whether Cas12a‐mediated ssDNA degradation occurs through defined mechanistic steps and how substrate chemistry and terminal structure influence cleavage kinetics and signal generation remain unclear. This limited mechanistic understanding restricts the rational design of optimized reporters for CRISPR‐based diagnostics.

Here, we hypothesized that Cas12a *trans*‐cleavage proceeds through defined directional substrate processing rather than indiscriminate stochastic degradation, and that elucidating these mechanisms would enable rational reporter engineering for improved diagnostic performance. Using a dedicated experimental framework to interrogate ssDNA cleavage, we found that Cas12a‐mediated *trans*‐cleavage comprises both endonucleolytic activity and a unidirectional 3′→5′ exonucleolytic trimming process (Figure 1a). We further demonstrated that terminal phosphate modification of ssDNA substrates accelerates substrate cleavage and modulates overall *trans*‐cleavage kinetics. Guided by these mechanistic insights, we designed ssDNA FQ reporters bearing 3′‐terminal phosphate modifications, which exhibited accelerated cleavage kinetics and enhanced fluorescence output (Figure 1b). By integrating these engineered reporters into the CRISPR–Cas12a platform, we developed Phos‐CRISPR (**Phos**phate‐engineered DNA reporters boost **CRISPR**), a rapid (20 min), sensitive, and specific assay that combines recombinase polymerase amplification (RPA) with CRISPR detection to enable nucleic acid analysis (Figure 1c). Validation using herpes simplex virus 1 (HSV‐1) clinical samples further revealed a clear relationship between fluorescence intensity and target concentration, supporting semi‐quantitative diagnostic capability. Together, these findings establish a mechanistically informed framework for CRISPR reporter engineering and introduce a generalizable design principle for improving the speed, sensitivity, and clinical performance of next‐generation CRISPR diagnostics.

**FIGURE 1:**
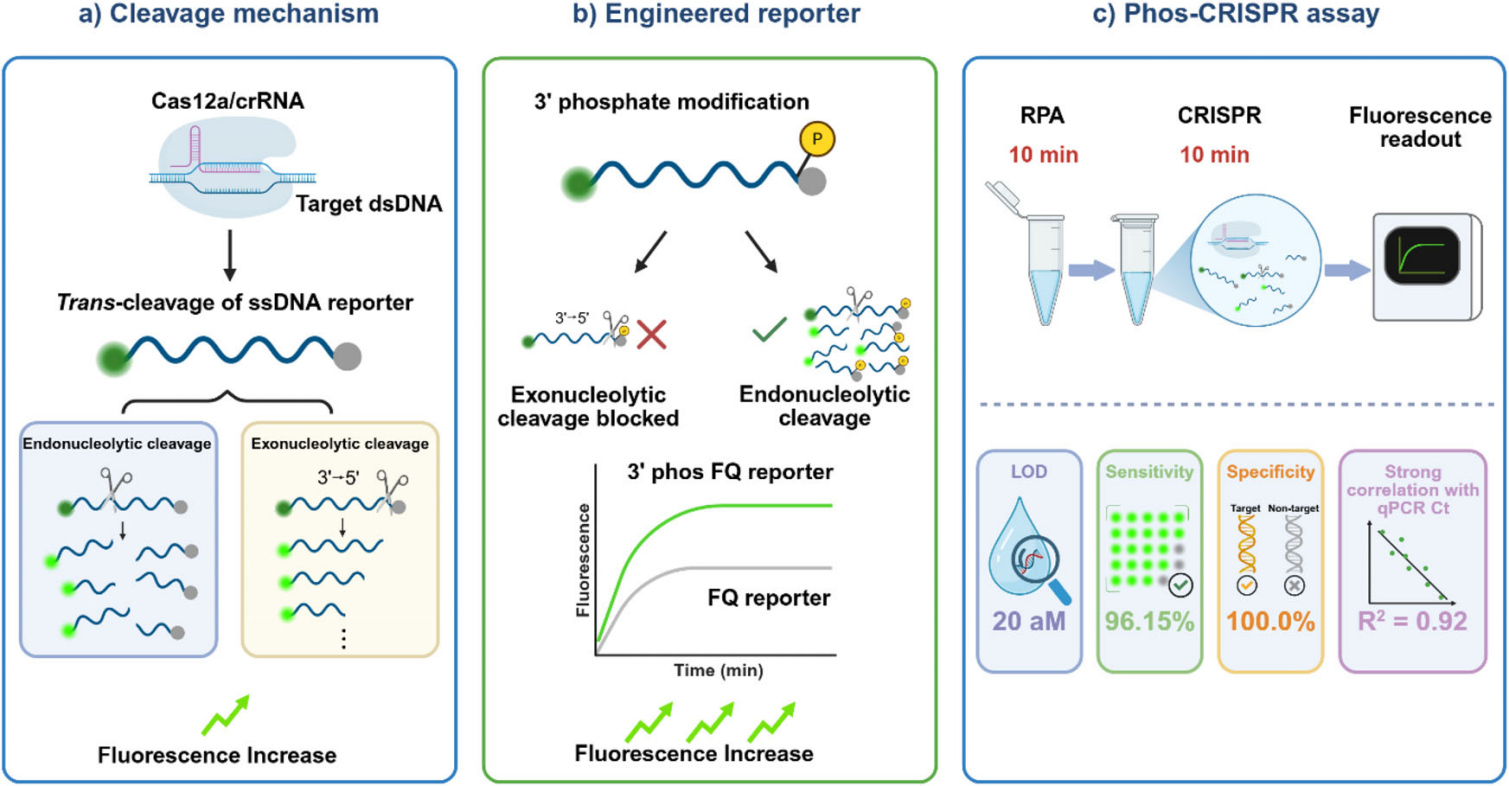
Phos‐CRISPR enables rapid, sensitive, and specific nucleic acid detection through phosphate‐modified ssDNA FQ reporters. a, Cas12a *trans*‐cleavage comprises both endonucleolytic cleavage and exonucleolytic trimming of ssDNA reporters. b, A terminal 3′‐phosphate modification accelerates substrate cleavage while favoring endonucleolytic processing, resulting in increased reporter turnover and fluorescence output. c, Incorporation of the engineered reporter into a CRISPR–Cas12a assay yields rapid Phos‐CRISPR, which combines 10‐min RPA amplification and 10‐min CRISPR detection, enabling highly sensitive and specific nucleic acid detection. (Created in BioRender. Yang, R. (2026) https://BioRender.com/ogw28qj).

## 2 Results

### 2.1 CRISPR‐Cas12a Exhibits Unidirectional Exonucleolytic *Trans*‐Cleavage Activity

We hypothesized that Cas12a‐mediated *trans*‐cleavage is not entirely indiscriminate but instead follows a defined cleavage pattern reflecting intrinsic substrate preferences and directional processing behavior (Figure 2a). To test this hypothesis, we performed CRISPR–Cas12a cleavage reactions and resolved the products using denaturing polyacrylamide gel electrophoresis.

**FIGURE 2:**
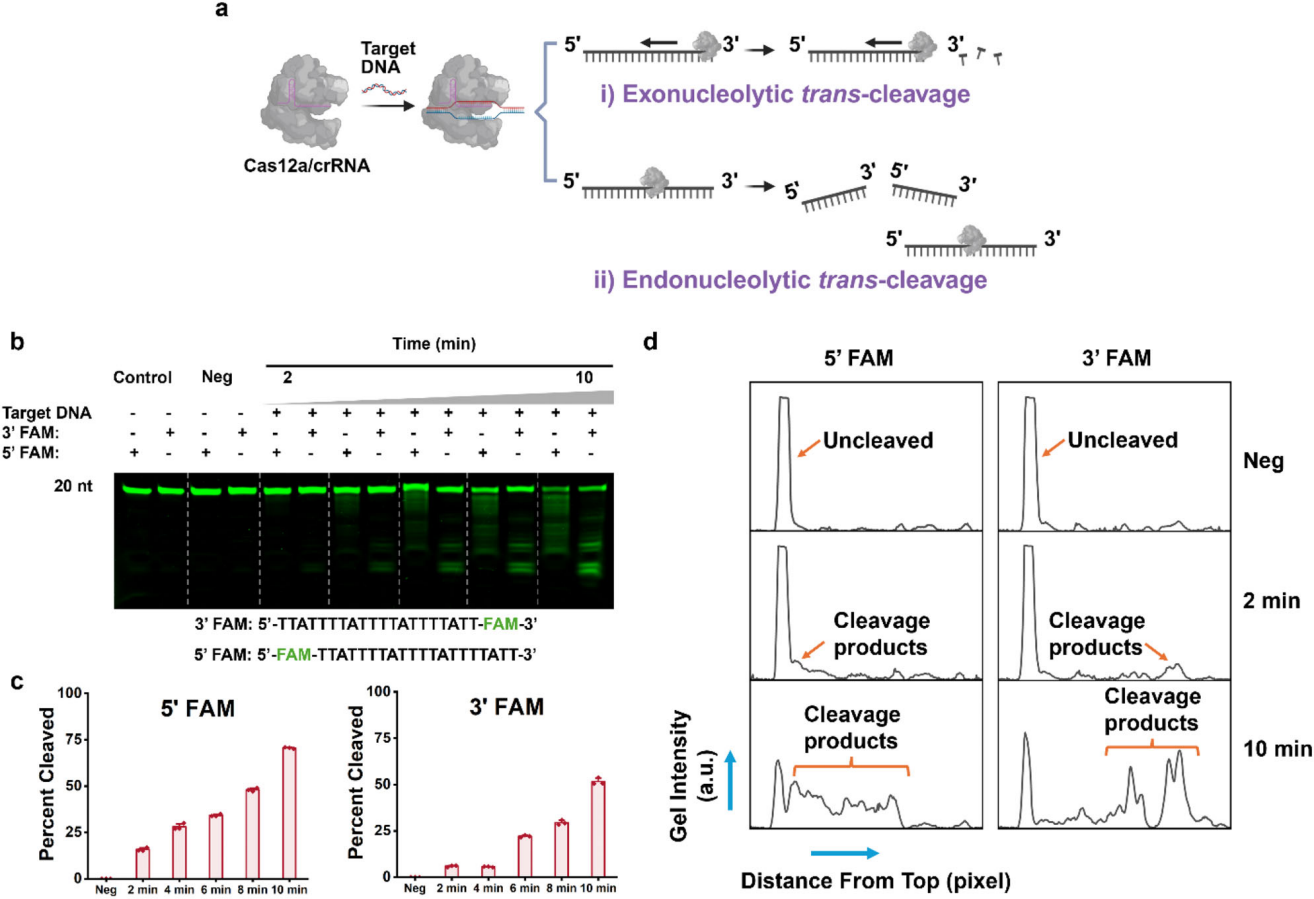
Characterization of CRISPR–Cas12a *trans*‐cleavage activity on ssDNA substrates. **a**, Schematic illustration of the proposed Cas12a *trans*‐cleavage comprising both endonucleolytic activity and a unidirectional 3′→5′ exonucleolytic trimming process. (Created in BioRender. Yang, R. (2026) https://BioRender.com/ogw28qj) **b**, Denaturing polyacrylamide gel electrophoresis analysis of *trans*‐cleavage products generated from 5′‐FAM‐ and 3′‐FAM‐labeled ssDNA substrates at different reaction times. Control lanes contained only intact ssDNA substrates, corresponding to the full‐length 20‐nt band. **c**, Comparison of cleavage efficiencies between ssDNA substrates labeled with 5′ FAM and 3′ FAM. **d**, Intensity plots of specific lanes (Neg, 2 min, 10 min) from background‐subtracted gel (Figure 2b) with ImageJ.

To enable direct visualization of degradation intermediates, we employed two 20‐nt ssDNA reporters labeled with a FAM fluorophore at either the 5′ or 3′ terminus. Following target activation of Cas12a, we quenched reactions at 2‐min intervals and analyzed time‐resolved fragmentation patterns by gel electrophoresis. In the absence of target DNA, the full‐length 20‐nt substrates remained intact. Upon activation, both 5′‐ and 3′‐FAM‐labeled reporters exhibited progressive degradation, as evidenced by a time‐dependent decrease in the full‐length band and the emergence of shorter fluorescent fragments (Figure 2b–d; Figures S1–S3).

Notably, the 5′‐FAM‐labeled substrate produced a pronounced ladder‐like pattern of progressively shorter fragments, consistent with stepwise degradation. In contrast, the 3′‐FAM‐labeled substrate showed rapid loss of the full‐length signal with substantially fewer detectable intermediate species. Quantification of cleavage efficiency based on disappearance of the full‐length 20‐nt band confirmed time‐dependent substrate degradation for both configurations (Figure 2c; Figure S2g–i), supporting progressive ssDNA processing upon Cas12a activation.

Comparison of fragment distributions further revealed a strong directional bias in substrate processing (Figure 2b,d). With the 5′‐FAM reporter, fluorescence was retained on progressively shorter fragments over time, generating a clear ladder pattern consistent with sequential degradation from the unlabeled 3′ terminus. By contrast, when the fluorophore was positioned at the 3′ terminus, fluorescence was rapidly lost, accompanied by reduced accumulation of intermediate fragments. The asymmetric fragmentation profiles observed between the two labeling configurations therefore indicate directional processing of ssDNA substrates. Together, these observations support a model in which Cas12a *trans*‐cleavage involves not only endonucleolytic cleavage but also unidirectional 3′→5′ exonucleolytic processing of ssDNA substrates.

### 2.2 Phosphate‐Modified ssDNA Boosts Cas12a *Trans*‐Cleavage Activity

Terminal or backbone modifications have been used to alter oligonucleotide nuclease susceptibility, including protection from exonuclease‐mediated degradation in serum or enzymatic systems [24, 25]. Previous studies have shown that terminal phosphate modifications can block exonucleolytic degradation in certain exonuclease systems by preventing processing from the modified end [26, 27]. Based on this observation, we hypothesized that terminal phosphorylation may accelerate substrate cleavage while favoring endonucleolytic processing (Figure 3a).

**FIGURE 3:**
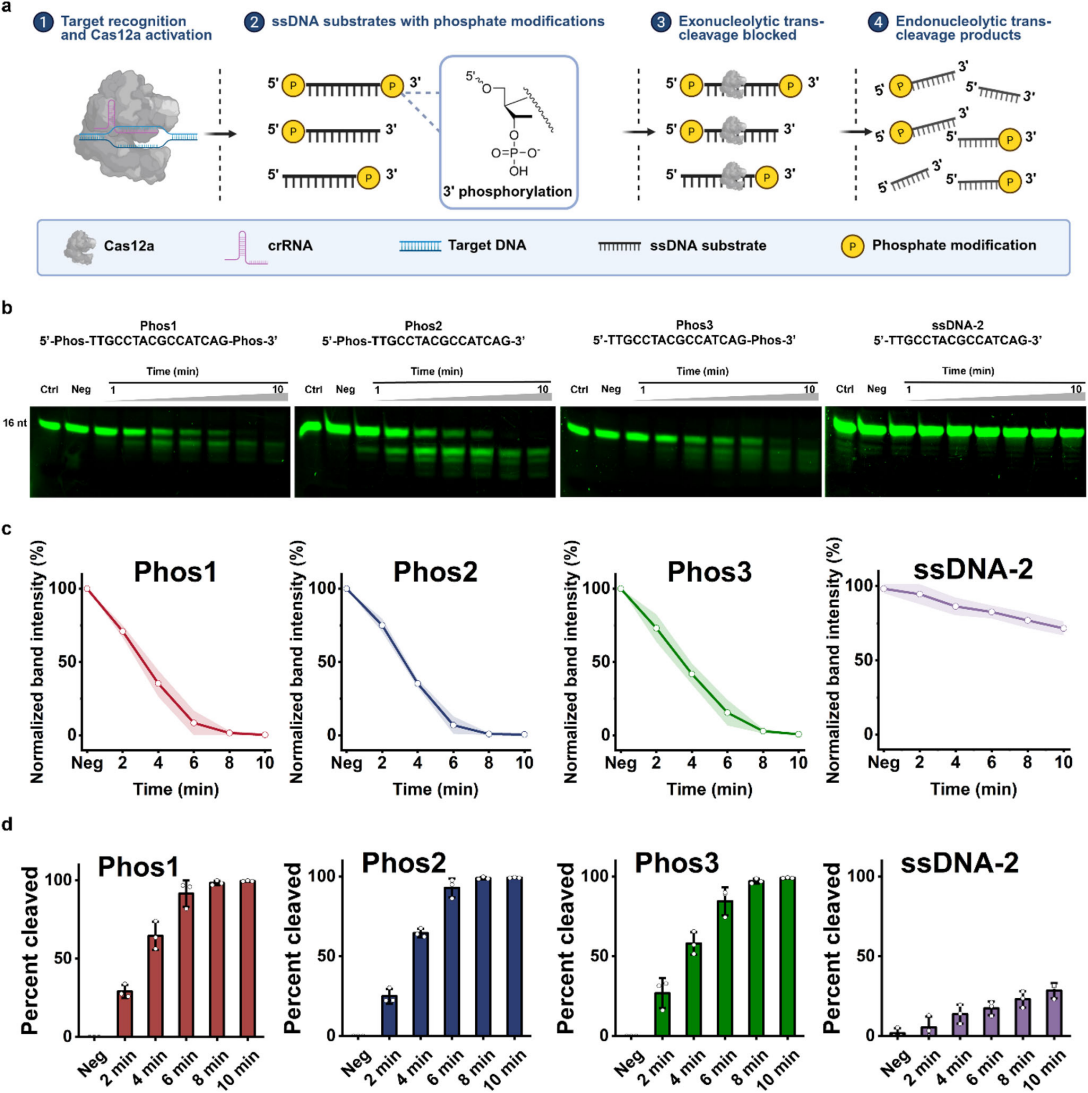
Characterization of CRISPR–Cas12a *trans*‐cleavage activity on phosphate‐modified ssDNA substrates. a, Schematic illustration of Cas12a *trans*‐cleavage on phosphate‐modified ssDNA substrates with exonucleolytic *trans*‐cleavage. (Created in BioRender. Yang, R. (2026) https://BioRender.com/ogw28qj) **b,** Denaturing gel electrophoresis analysis of *trans*‐cleavage products generated from phosphate‐modified ssDNA substrates and unmodified ssDNA substrates. Control lanes contained only ssDNA substrate corresponding to the full‐length ssDNA band. All substrates were used at the same initial substrate concentration of 1 µm. Gels for the different substrates were imaged separately. **c,** Comparison of normalized band intensities of phosphate‐modified ssDNA substrates and unmodified ssDNA substrate control over time. **d,** Comparison of cleavage percentages of three phosphate‐modified ssDNA substrates and one unmodified ssDNA substrate control at different reaction time points. Data are presented as mean ± s.d. (n = 3).

To test this hypothesis, we designed three ssDNA substrates bearing phosphate modifications at different termini (Phos1: 5′‐ and 3′‐phosphate; Phos2: 5′‐phosphate only; Phos3: 3′‐phosphate only) and compared them with an unmodified control (ssDNA). Denaturing gel electrophoresis showed that all phosphate‐modified ssDNA substrates exhibited predominantly discrete cleavage products with markedly reduced progressive shortening over time, whereas the unmodified ssDNA control exhibited a ladder‐like fragmentation pattern (Figure 3b). Quantitative analysis based on normalized full‐length band intensity and full‐length substrate depletion confirmed more rapid cleavage of the phosphate‐modified substrates (Figure 3c,d; Figure S4). Phosphate‐modified ssDNA substrates exhibited markedly accelerated cleavage kinetics, reaching ∼50–70% cleavage within 4–5 min and >90% cleavage by 10 min, respectively (Figure 3d). In contrast, the unmodified ssDNA substrate showed substantially slower cleavage, achieving only ∼30% cleavage after 10 min. These results demonstrate that terminal phosphorylation accelerates Cas12a‐mediated trans‐cleavage of ssDNA substrates, enabling faster substrate consumption and potentially contributing to the enhanced fluorescence output.

Collectively, these findings reveal that terminal phosphate modification reprograms Cas12a‐mediated ssDNA processing, promoting rapid and efficient substrate turnover while favoring endonucleolytic cleavage.

### 2.3 Phosphate‐Modified ssDNA Reporters Improve CRISPR‐Based Nucleic Acid Detection

To investigate how terminal chemistry influences Cas12a‐mediated signal generation, we designed three ssDNA FQ reporters bearing phosphate modifications at distinct termini (Figure 4a): dual‐terminal phosphorylation, 5′‐phosphate only, and 3′‐phosphate only. We included an unmodified ssDNA as a control. Because the vendor was unable to synthesize phosphate‐modified ssDNA FQ reporters shorter than 10 nt, we selected 10‐nt phosphate‐modified ssDNA FQ reporters to evaluate and compare CRISPR‐based nucleic acid detection.

**FIGURE 4:**
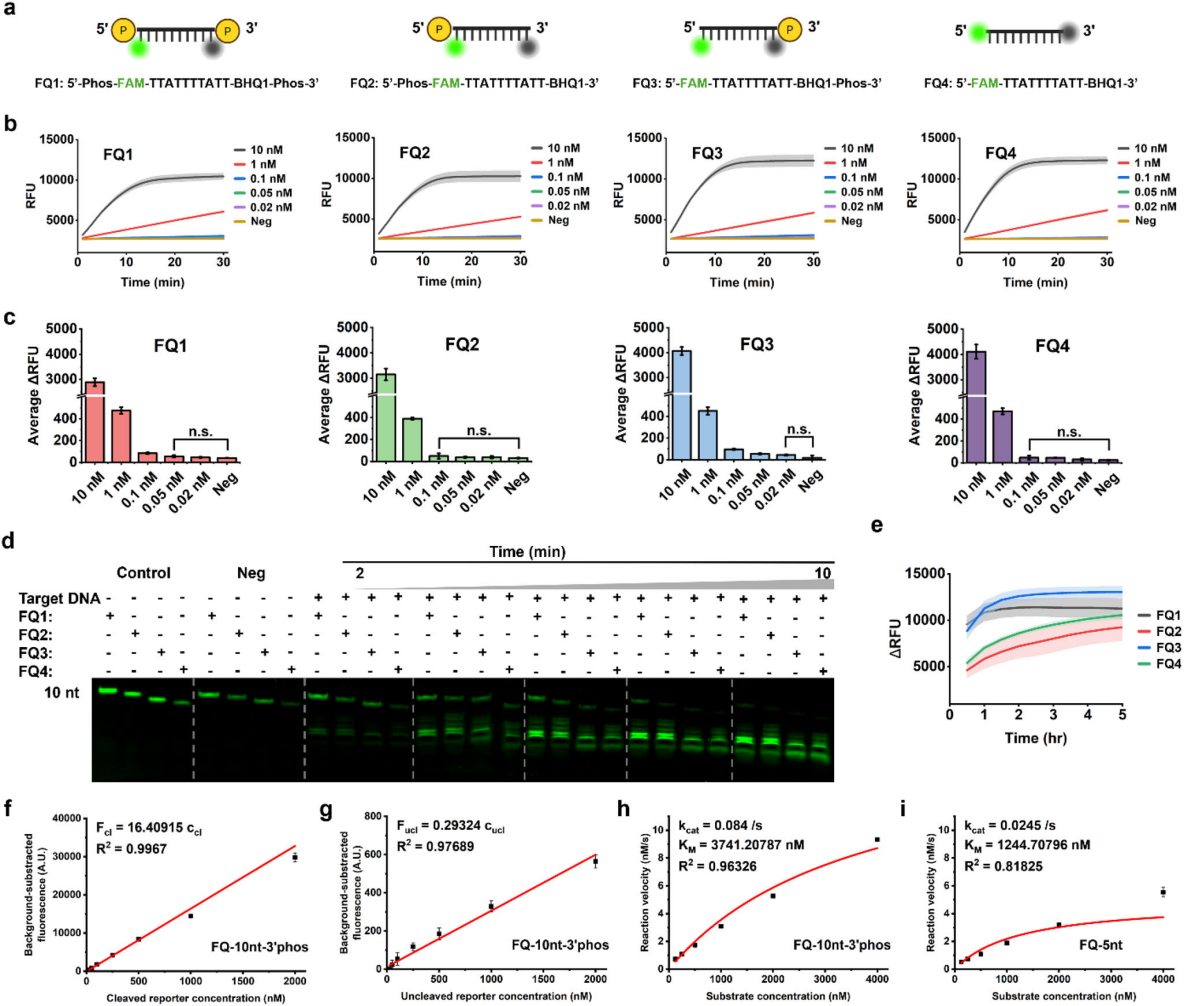
Evaluation of phosphate‐modified ssDNA FQ reporters for CRISPR–Cas12a nucleic acid detection and kinetic analysis. a, Schematic illustration of three phosphate‐modified ssDNA FQ reporters (FQ1–FQ3) and one unmodified ssDNA FQ reporter (FQ4). (Created in BioRender. Yang, R. (2026) https://BioRender.com/ogw28qj) **b**, Fluorescence kinetics of phosphate‐modified and unmodified ssDNA FQ reporters at different target concentrations. Data are presented as mean ± s.d. (n = 3). **c**, Mean fluorescence intensity increase (difference between initial and endpoint fluorescence) at each target concentration (mean ± s.d.) after a 5‐min reaction. Statistical significance was assessed by t‐test between each target concentration and the negative control. **d**, Denaturing gel electrophoresis analysis of *trans*‐cleavage products generated from phosphate‐modified and unmodified ssDNA FQ reporters. Control lanes contained intact reporters only, corresponding to the full‐length 10‐nt band. **e**, Fluorescence increase following 5 h DNase I digestion of phosphate‐modified and unmodified ssDNA FQ reporters. Data are presented as mean ± s.d. (n = 3). **f**, Background‐subtracted fluorescence (F_cl_) as a function of cleaved reporter concentration (c_cl_). **g**, Background‐subtracted fluorescence (F_ucl_) as a function of uncleaved reporter concentration (c_ucl_). **h**, Michaelis–Menten kinetics of ssDNA‐activated *trans*‐cleavage by CRISPR‐Cas12a using the 3’phos FQ reporter. **i**, Michaelis–Menten kinetics of ssDNA‐activated *trans*‐cleavage by CRISPR‐Cas12a using the unmodified FQ reporter. Data are presented as mean ± s.d. (n = 3). Red solid lines represent nonlinear fits to the Michaelis–Menten model, and fitted kinetic parameters are indicated in each panel.

We first evaluated real‐time fluorescence recovery during CRISPR–Cas12a reactions across a range of target concentrations (Figure 4b). Reporters bearing terminal phosphate modifications exhibited enhanced signal amplification relative to the unmodified control (Figure S5). At high target concentration (10 nm), all reporters generated strong fluorescence signals; however, phosphate‐modified reporters showed faster signal accumulation and higher plateau intensities, particularly for the 3′‐phosphorylated design. At lower target concentrations, signal separation decreased, although the 3′‐phosphorylated reporter maintained the highest relative signal intensity. Quantification of fluorescence intensity at the 5‐min time point further confirmed these trends (Figure 4c), with the 3′‐phosphorylated reporter exhibiting the best detection performance and the lowest limit of detection (0.05 nm) without RPA pre‐amplification. This enhanced performance may be attributed to reduced susceptibility of the ssDNA reporter to exonucleolytic degradation following terminal 3′‐phosphate modification, consistent with previous studies [26, 27]. Importantly, fluorescence generation in CRISPR assays primarily results from the initial cleavage of intact, full‐length FQ reporters, which separates the fluorophore from the quencher. Therefore, progressive exonucleolytic trimming of an already cleaved reporter does not constitute an additional productive fluorescence‐generation event. In addition, the 5′‐phosphorylated reporter enabled detection of 0.1 nm DNA target, whereas the unmodified control did not produce a detectable signal within 15 min (Figure S6).

To relate fluorescence output to cleavage behavior, we analyzed reporter degradation by denaturing gel electrophoresis (Figure 4d; Figures S7–S9). All phosphate‐modified reporters exhibited accelerated substrate consumption compared with the unmodified control, consistent with enhanced *trans*‐cleavage kinetics. However, fluorescence output did not scale directly with cleavage rate, indicating that terminal phosphorylation and reporter architecture jointly influence fluorescence recovery in addition to enzymatic cleavage. To further decouple cleavage kinetics from intrinsic reporter signal properties, we assessed maximal fluorescence recovery independent of Cas12a activity using prolonged DNase I digestion (Figure 4e; Figure S10). Terminal phosphate modification significantly affected ultimate fluorescence release capacity, confirming that differences in endpoint fluorescence reflect both enzymatic cleavage efficiency and intrinsic reporter fluorescence recovery behavior. Collectively, these results demonstrate that the 3′‐phosphate‐modified FQ (3’Phos FQ) reporter provides the optimal balance between rapid *trans*‐cleavage kinetics and efficient fluorescence recovery, resulting in improved sensitivity and higher endpoint signal output compared with the unmodified reporter.

To quantitatively evaluate the effect of terminal phosphate modification on Cas12a *trans*‐cleavage kinetics, we first established fluorescence calibration curves for both cleaved and uncleaved reporters (Figure 4f,g). We generated calibration curves for the 3’phos FQ reporter following complete digestion with DNase I at 37°C for 10 h and obtained calibration curves for the FQ reporter as previously described [28]. Fluorescence intensity showed a strong linear correlation with the concentration of cleaved reporter (R^2^ = 0.9967, slope ≈ 16.409 A.U. nM^−^^1^), enabling accurate conversion of fluorescence units to molar concentrations of cleavage products. By contrast, uncleaved reporters generated minimal fluorescence signals with a slope of only 0.293 A.U. nM^−^^1^, approximately 56‐fold lower than that of the fully cleaved reporter.

Michaelis–Menten analysis revealed distinct kinetic profiles for the 3’phos FQ and conventional FQ reporters (Figure 4h,i). The 3’phos FQ reporter exhibited a substantially higher turnover rate (k_cat_ = 0.084 s^−^^1^) than the FQ reporter (k_cat_ = 0.0245 s^−^^1^), indicating accelerated *trans*‐cleavage kinetics. This enhanced catalytic turnover was accompanied by an increased Michaelis constant (K_M_ = 3,741 nm versus 1244 nm), suggesting reduced substrate affinity. We next compared the catalytic efficiencies (k_cat_/K_M_) of Cas12a *trans*‐cleavage using the two reporter substrates and ssDNA activators. The maximum catalytic efficiency for the 3’phos FQ reporter reached 2.26 × 10^4^ M^−^^1^ s^−^^1^, compared with 1.97 × 10^4^ M^−^^1^ s^−^^1^ for the FQ reporter. Table S1 summarizes these kinetic parameters alongside those reported in previous studies. Collectively, these results demonstrate that the 3’phos FQ reporter supports more rapid fluorescence signal generation than the conventional FQ reporter, highlighting its improved performance for CRISPR–Cas12a‐based molecular detection.

### 2.4 HSV‐1 DNA Detection by Phos‐CRISPR Using 3′Phos ssDNA FQ Reporters

Owing to its enhanced *trans*‐cleavage kinetics and improved fluorescence recovery, we selected the 3’phos FQ reporter to develop a rapid and sensitive Phos‐CRISPR assay for HSV‐1 DNA detection by combining RPA with CRISPR (Figure 5a). Kinetic characterization demonstrated that the 3’phos FQ reporter exhibited faster cleavage dynamics and more rapid fluorescence signal accumulation than the conventional reporter. Leveraging these improved reaction properties, we shortened the overall assay workflow to 20 min, including 10 min for RPA and 10 min for CRISPR–Cas12a detection. This resulted in an approximately threefold reduction in assay time relative to conventional CRISPR assay workflows, which generally require 20 min of amplification and 30–60 min of CRISPR incubation, corresponding to a total assay time of 50–80 min [29, 30].

**FIGURE 5:**
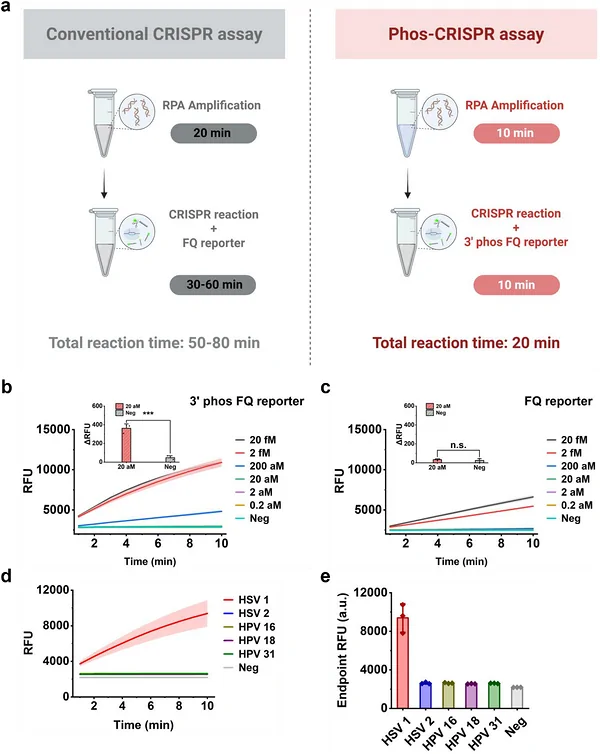
Performance assessment of the Phos‐CRISPR assay using 3’phos FQ reporters. **a**, Comparison of detection times between Phos‐CRISPR and conventional CRISPR assays using the 3′‐phosphate FQ reporter and the unmodified FQ reporter, respectively. (Created in BioRender. Yang, R. (2026) https://BioRender.com/ogw28qj). **b**, Real‐time fluorescence kinetics of the Phos‐CRISPR assay using a 3′ phos FQ reporter. Inset: Comparison of endpoint fluorescence signal enhancement between the 20 aM target sample and the negative control after a 10 min Phos‐CRISPR reaction. **c**, Real‐time fluorescence kinetics of the conventional CRISPR assay using an unmodified FQ reporter. Inset: Comparison of endpoint fluorescence signal enhancement between the 20 aM target sample and the negative control after a 10 min conventional CRISPR reaction. **d**, Fluorescence kinetics of the Phos‐CRISPR assay using 3’phos FQ reporters for HSV‐1 DNA specificity evaluation. HSV‐1, HSV‐2, HPV‐16, HPV‐18, and HPV‐31 DNA targets were tested. **e**, Endpoint fluorescence detection results of Phos‐CRISPR for HSV‐1, HSV‐2, HPV‐16, HPV‐18, and HPV‐31 DNA targets. Data are presented as mean ± s.d. (n = 3).

Real‐time fluorescence analysis revealed markedly enhanced detection sensitivity with the 3′‐phosphate FQ reporter relative to the conventional FQ reporter (Figure 5b,c; Figure S11). Phos‐CRISPR generated substantially higher fluorescence signals at identical target concentrations, whereas the conventional CRISPR assay showed slower signal accumulation and reduced discrimination between low‐concentration targets and the negative control. We next quantified fluorescence signal enhancement at 20 aM relative to the negative control. The 3’phos FQ reporter produced statistically significant signal separation (***P ≤ 0.001), whereas the FQ reporter showed no significant difference (insets, Figure 5b,c). Collectively, the enhanced reaction kinetics and improved fluorescence recovery of the 3’phos FQ reporter enabled reliable signal accumulation under shortened assay conditions, achieving a limit of detection of 20 aM (∼12 DNA copies/µL).

We next assessed the detection specificity of the optimized Phos‐CRISPR assay using the 3’phos FQ reporter. We tested HSV‐1 as the target, while HSV‐2 and multiple HPV subtypes (HPV‐16, HPV‐18, and HPV‐31) served as non‐target controls. Only HSV‐1 DNA generated a strong fluorescence signal; HSV‐2, HPV subtypes, and the negative control exhibited minimal signal increases. Both real‐time fluorescence curves (Figure 5d) and endpoint fluorescence analysis (Figure 5e) demonstrated significant discrimination between HSV‐1 and all non‐target samples. These results indicate that the shortened assay maintains high specificity without detectable cross‐reactivity.

### 2.5 Clinical Testing and Validation

Finally, we evaluated the clinical performance of the Phos‐CRISPR assay incorporating the 3’phos FQ reporter for HSV‐1 DNA detection. We analyzed a total of 37 de‐identified clinical swab samples using the optimized workflow (Figure 6a). We first extracted HSV‐1 DNA using a commercial DNA extraction kit, followed by Phos‐CRISPR detection with real‐time fluorescence readout. To ensure measurement reliability, we tested each sample in triplicate.

**FIGURE 6:**
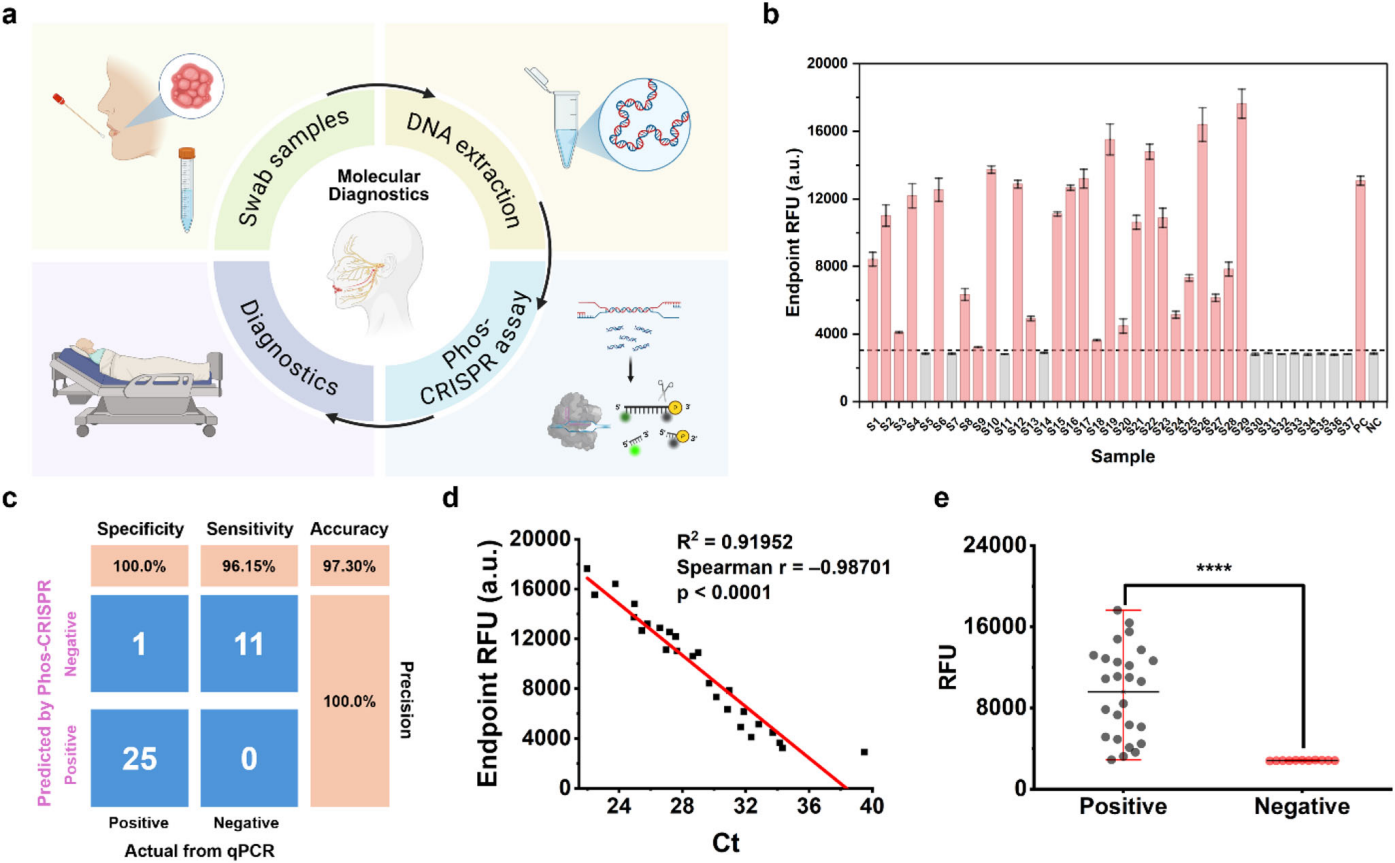
Clinical validation of the Phos‐CRISPR assay for HSV‐1 detection using 3’phos FQ reporters. **a**, Schematic illustration of the rapid and sensitive Phos‐CRISPR detection workflow employing 3’phos FQ reporters for clinical applications. (Created in BioRender. Yang, R. (2026) https://BioRender.com/ogw28qj) **b**, Endpoint fluorescence intensities for individual clinical samples using the Phos‐CRISPR assay. The dashed line indicates the positivity threshold. **c**, Confusion matrix analysis comparing Phos‐CRISPR assay results with qPCR results for 37 clinical samples, including 26 qPCR‐positive and 11 qPCR‐negative samples. **d**, Correlation analysis between Phos‐CRISPR endpoint fluorescence signals and qPCR cycle threshold (Ct) values. **e**, Dot plot showing HSV‐1 detection using the Phos‐CRISPR assay with the 3’phos FQ reporter. Individual dots represent sample‐level means calculated from three replicates, while horizontal lines denote the group averages for negative and positive samples, respectively. **** p = 0.000016.

Endpoint fluorescence values for all clinical samples are shown in Figure 6b. We classified samples exceeding the threshold (defined as the mean of the negative control + 5 s.d.) as positive, whereas samples below the threshold were considered negative. Using this criterion, we identified 25 samples as HSV‐1 positive and 12 as negative. The real‐time fluorescence kinetic profiles further confirmed the detection trends observed across the clinical samples, with positive samples showing progressive fluorescence increases and negative samples remaining near background levels (Figure S12). In parallel, we validated all samples using gold‐standard quantitative PCR (qPCR). Comparison of Ct values and detection outcomes (Figure 6c; Table S2) showed that the Phos‐CRISPR assay with 3’phos FQ correctly identified 25 of 26 qPCR‐positive samples, corresponding to a sensitivity of 96.15%. No false‐positive results were observed among qPCR‐negative samples, yielding a specificity of 100.0% and an overall accuracy of 97.30%.

The relationship between Phos‐CRISPR endpoint fluorescence intensity and qPCR Ct values is shown in Figure 6d. Notably, we observed a strong inverse correlation (Spearman r = −0.9871, p < 0.0001), indicating that higher viral loads (lower Ct values) generate stronger fluorescence signals. This semi‐quantitative capability likely arises from the shortened RPA amplification time used in the Phos‐CRISPR assay together with the enhanced fluorescence output provided by the 3′‐phosphate FQ reporter. Consistently, the dot plot in Figure 6e demonstrates clear separation between HSV‐1‐positive and HSV‐1‐negative clinical samples based on endpoint fluorescence intensity. Positive samples exhibited significantly higher fluorescence signals with a broad distribution, reflecting variable viral loads across clinical specimens, whereas negative samples remained tightly clustered near baseline with minimal background variation. Mean fluorescence intensity was significantly elevated in the positive samples compared with the negative samples (****, p = 0.000016), supporting the assay's strong specificity and robust clinical performance. Collectively, these results demonstrate that the Phos‐CRISPR assay incorporating the 3’phos FQ reporter enables simple, accurate, and robust detection of HSV‐1 in clinical specimens and highlights its strong potential for rapid point‐of‐care diagnostic applications.

## 3 Discussion

CRISPR–Cas12a has been previously shown to exhibit sequence‐specific *cis*‐cleavage of target dsDNA, as well as indiscriminate *trans*‐cleavage of non‐target ssDNA [31, 32, 33]. Here, we systematically investigated the *trans*‐cleavage mechanism of Cas12a and demonstrated that it displays both endonucleolytic and unidirectional exonucleolytic activities on ssDNA substrates. Time‐resolved gel electrophoresis and cleavage product profiling revealed a characteristic ladder‐like pattern consistent with progressive and sequential ssDNA degradation. Using FAM‐labeled substrates, we further observed evidence supporting unidirectional 3′→5′ exonucleolytic trimming in addition to endonucleolytic cleavage.

Guided by these mechanistic insights, we introduced terminal phosphate modifications into ssDNA substrates and FQ reporters. Both unlabeled ssDNA substrates and FQ reporters bearing 3′‐phosphate modifications exhibited accelerated degradation kinetics compared with unmodified controls, with more than 90% of substrates cleaved within 10 min. Although terminal phosphate modification could, in principle, influence fluorescence recovery, the 3′phos FQ reporters nevertheless showed the most efficient signal generation while maintaining robust cleavage kinetics.

Leveraging this improved reporter performance, we established a rapid Phos‐CRISPR diagnostic workflow that reduced the assay time by threefold. The RPA step was shortened to 10 min, approximately half of the conventional duration, while CRISPR detection was reduced to 10 min compared with commonly used 30–60 min protocols [18, 34]. Using the optimized 3′‐phosphorylated ssDNA FQ reporter, the assay achieved a limit of detection of 20 aM. Clinical validation further demonstrated reliable performance, with 96.15% sensitivity, 100.0% specificity, and 97.30% overall accuracy, together with a strong correlation to qPCR Ct values.

Collectively, these results indicate that terminal phosphate modification enhances Cas12a‐mediated *trans*‐cleavage kinetics, fluorescence output, and overall diagnostic sensitivity, enabling rational translation of mechanistic insights into reporter engineering. However, the structural basis of substrate engagement and terminal recognition by Cas12a remains unresolved. Existing structural and biochemical studies have clarified key aspects of PAM‐dependent target recognition, *cis*‐cleavage, and RuvC‐dependent activation of *trans*‐cleavage, but they do not fully explain how activated Cas12a accommodates chemically modified ssDNA substrates during collateral cleavage [33, 35]. High‐resolution structural studies will be needed to elucidate how terminal phosphate modifications alter enzyme–substrate interactions and modulate endonucleolytic and exonucleolytic activities. In addition, systematic investigation of alternative chemical modifications to ssDNA reporters will further improve understanding of how reporter chemistry influences cleavage dynamics and fluorescence signal generation.

Overall, our findings highlight that Cas12a *trans*‐cleavage dynamics are not inherently indiscriminate but can instead be modulated through rational reporter design. By tuning reporter terminal chemistry, cleavage kinetics and signal output can be engineered without altering the Cas12a protein itself, providing a simple and cost‐effective strategy to improve CRISPR‐based diagnostics. Compared with protein engineering approaches, reporter optimization is experimentally straightforward and readily implementable, yet yields substantial improvements in sensitivity, speed, and accuracy, thereby enhancing suitability for point‐of‐care applications.

## 4 Methods

### 4.1 Ethical Statement

In this study, all clinical swab samples were de‐identified prior to analysis and provided by the Clinical Microbiology Laboratory under a protocol approved by the Institutional Review Board (IRB) at the University of Connecticut Health Center (IRB# 22–215‐1). The study was approved by the IRB, with a waiver of informed consent, because only de‐identified clinical samples without patient‐identifiable information were used.

### 4.2 Materials

All oligonucleotides used in this study, listed in Table S3, were obtained from Integrated DNA Technologies Inc. (IDT, USA). Nuclease‐free water, Lba Cas12a (Cpf1), and 10× NEBuffer r2.1 were purchased from New England BioLabs (MA, USA). The TwistAmp Basic RPA kit and the GoTaq qPCR kit were purchased from Fisher Scientific Company, LLC (GA, USA). Urea was obtained from Sigma‐Aldrich (MO, USA). The 10× TBE buffer, 40% acrylamide/bis solution (19:1), TEMED, and ammonium persulfate (APS) were purchased from Bio‐Rad (CA, USA). The 2× Gel Loading Buffer II (Denaturing PAGE) and SYBR Gold Nucleic Acid Gel Stain (10,000× concentrate in DMSO) were purchased from Thermo Fisher Scientific (MA, USA). The DNeasy Blood and Tissue Kit was obtained from QIAGEN (MD, USA). DNase I and DNase I Buffer were obtained from Thermo Fisher Scientific.

### 4.3 CRISPR–Cas12a *Trans*‐Cleavage Assay

To evaluate the *trans*‐cleavage activity of CRISPR–Cas12a, a 4× Cas12a/crRNA ribonucleoprotein complex was pre‐assembled by mixing 0.768 µL Lba Cas12a (100 µm), 9.6 µL T1 crRNA (10 µm), and 86 µL diluent. The mixture was incubated at 37°C for 30 min to facilitate complex formation. The final CRISPR reaction was carried out in a total volume of 10 µL, consisting of 2.5 µL 4× Cas12a/crRNA complex, 1 µL 10× NEBuffer r2.1, 1 µL 10 µm ssDNA reporter, 4.5 µL nuclease‐free water, and 1 µL T1 DNA target for positive controls or 1 µL nuclease‐free water for negative controls. Reactions were incubated at 37°C for 30 min and deactivated at 95°C for 2 min.

### 4.4 Gel Electrophoresis

To visualize the CRISPR–Cas12a *trans*‐cleavage activities with different ssDNA reporters, 10% denaturing polyacrylamide gel electrophoresis (PAGE) was performed. For gel preparation, 4.8 g urea was dissolved in 10 mL 40% acrylamide/bis (19:1) and 2 mL 10× TBE buffer, and adjusted to 20 mL with deionized water. Polymerization was initiated by adding 8 µL TEMED and 66 µL 30% APS. The solution was then transferred into the gel‐casting chamber.

The CRISPR–Cas12a reactions were initiated simultaneously by brief centrifugation after addition of either DNA target or nuclease‐free water. Reactions were terminated at defined time points by mixing 10 µL CRISPR product with 20 µL 2× Gel Loading Buffer II (Denaturing PAGE), and tubes were immediately transferred on ice. After the completion of all CRISPR‐Cas12a reactions, the mixture was incubated at 95°C for 10 min. Then, 10 µL of the mixture was loaded onto the gel. In addition, 1 µm ssDNA reporter mixed with 2× Gel Loading Buffer II was added to the first lane as a reference control. Electrophoresis was conducted at a constant 20 mA for 1.5 h. For reactions using ssDNAs lacking FAM labels, gels were stained with SYBR Gold Nucleic Acid Gel Stain for 10 min. Gels were imaged using the ChemiDoc MP imaging system (Bio‐Rad, CA, USA; RRID: SCR_019684).

### 4.5 Gel Image Analysis

Background‐subtracted gel images were analyzed using ImageJ (RRID: SCR_003070). Lane intensity profiles were obtained by averaging pixel intensities across the lane width and plotted. Full‐length band intensities were normalized (peak intensity at each time point divided by the peak intensity of the negative control) and plotted versus time. Percent cleavage of the full‐length ssDNA substrates was calculated based on band densitometry.

$$\begin{array}{cc} & \mathrm{Percent}\;\mathrm{cleavage}\;\;\left(\%\right)\\ & \;=100\%-\frac{\mathrm{Full}\;\mathrm{length}\;\mathrm{band}\;\mathrm{intensity}}{\mathrm{Negative}\;\mathrm{control}\;\mathrm{band}\;\mathrm{intensity}}\times 100\% \end{array}$$

### 4.6 Cleavage Reactions of Phosphate‐modified ssDNA Reporters in CRISPR and DNase I

The cleavage reaction was conducted in a final volume of 10 µL, consisting of 2.5 µL 4× Cas12a/crRNA complex, 1 µL 10× NEBuffer r2.1, 1 µL 10 µm phosphate‐modified ssDNA reporter, 4.5 µL nuclease‐free water, and 1 µL T1 DNA target for positive controls (or 1 µL nuclease‐free water for negative controls). Real‐time fluorescence signals were measured every minute using a CFX96 Touch Real‐Time PCR Detection System (Bio‐Rad, CA, USA; RRID: SCR_018064). Reaction products obtained at defined time points were resolved by 10% denaturing PAGE for normalized intensity and percent cleavage analysis.

To evaluate the fluorescence intensity of each phosphate‐modified ssDNA reporter and the conventional FQ ssDNA reporter, a DNase I cleavage assay was performed in a total volume of 20 µL. DNase I (1 µL), 10 µm reporter (2 µL), 10× DNase I Buffer (2 µL), and nuclease‐free water (15 µL) were mixed and incubated at 37°C for 5 h. At the end of the reaction, the fluorescence intensities for all reporters reached a steady state.

### 4.7 Michaelis–Menten Kinetics Measurement

The Michaelis–Menten kinetics were determined following the protocol reported previously [28, 36, 37]. Experiments were performed as follows: 1) 4× Cas12a/crRNA complex (including 200 nM Cas12a and 250 nM T1 crRNA), 10× NEBuffer r2.1, and nuclease‐free water were incubated with a 10‐fold molar excess of T1 DNA target (2 µm) at 37°C for 30 min. 2) The *trans*‐cleavage assay was initiated by incubating the mixture from step 1 with 125, 250, 500 nm, 1, 2, or 4 µm ssDNA reporters at 37°C. Fluorescence signals were measured at 30‐s intervals, and the initial reaction velocities were calculated from the first 5 min of the fluorescence readouts.

Calibration of fluorescence versus substrate concentration was performed using reporter concentrations ranging from 25 nm to 2 µm. FQ‐10nt‐3’‐phosphate reporters were cleaved by DNase I at 37°C for 10 h. FQ reporters were cleaved by AsCas12a Ultra at 37°C for 10 h according to our previous study [28]. At the end of the reaction, the fluorescence intensities for all reporters reached a steady state. Linear regression was performed between background‐subtracted fluorescence *F_cl_* and cleaved reporter concentration *c_cl_*, yielding *F_cl_* =  16.40915*c_cl_*, where *c_cl_* is in nM and *F_cl_* is in arbitrary units (AU). Calibration of fluorescence versus uncleaved reporter concentration was performed using the same protocol but without DNase I. Linear regression between background‐subtracted fluorescence *F_ucl_* and uncleaved reporter concentration *c_ucl_* yielded *F_ucl_* =  0.29324 *c_ucl_*, where *c_ucl_* is in nM and *F_ucl_* is in AU.

Reaction velocities were estimated using a mathematical model derived from the calibration curves. The total background‐subtracted fluorescence *F*(*t*) is the sum of contributions from cleaved (*F_cl_* (*t*)) and uncleaved (*F_ucl_* (*t*)) reporters:

(1) $$F\left(t\right)=F_{cl}\left(t\right)+F_{ucl}\left(t\right)$$

Linear regression yields:

(2) $$F\;\left(t\right)=16.40915c_{cl}\left(t\right)+0.29324\;c_{ucl}\left(t\right)$$

Given that *c_cl_* (*t*) +  *c_ucl_* (*t*) =  *c*_0_, where *c*_0_ is the initial uncleaved reporter concentration, Equation (2) can be reformulated as:

(3) $$\begin{array}{cc} F\;\left(t\right) & =16.40915c_{cl}\left(t\right)+0.29324\;\;\left(c_{0}-c_{cl}\left(t\right)\right)\\ & =16.11591c_{cl}\left(t\right)+0.29324\;c_{0} \end{array}$$

The reaction velocity, defined as *dc_cl_*/*dt* (nM s^−1^), was obtained by differentiating Equation (3) with respect to time:

(4) $$\frac{dc_{cl}}{dt}=\frac{1}{16.11591}\;\times \frac{dF}{dt}$$

Initial velocities were fitted to the Michaelis–Menten equation:

(5) $$v=k_{cat}E_{0}\frac{\left[S\right]}{K_{M}+\left[S\right]}$$

Here, *k_cat_* is the catalytic turnover rate, *E*_0_ is the activated enzyme concentration (200 nm), [*S*] is the substrate concentration, and *K_M_* is the Michaelis–Menten constant.

### 4.8 RPA/CRISPR–Cas12a Assay Using Phosphate‐modified ssDNA Reporters

To establish an RPA/CRISPR–Cas12a assay incorporating phosphate‐modified ssDNA reporters for HSV‐1 DNA detection, a two‐step workflow was performed. HSV‐1 DNA templates were first pre‐amplified using an RPA kit according to the manufacturer's instructions. The 50 µL RPA reaction mixture consisted of 29.5 µL rehydration buffer, 2.4 µL each of 10 µm forward and reverse primers, 2.5 µL MgOAc, 12.2 µL nuclease‐free water, and 1 µL HSV‐1 DNA template for positive controls or nuclease‐free water for negative controls. RPA reactions were incubated at 37°C for 10 min. Subsequently, 1 µL of the amplified product was transferred into the CRISPR reaction mixture containing 3′‐phosphate‐modified FQ reporters and incubated at 37°C for an additional 10 min. The reaction temperature of 37°C was used for both the RPA and CRISPR reactions, based on previously reported literature and the manufacturer's recommended conditions [2, 6, 10]. Real‐time fluorescence signals were recorded at 1 min intervals using a CFX96 Touch Real‐Time PCR Detection System (Bio‐Rad, CA, USA; RRID: SCR_018064). HSV‐1 DNA targets ranging from 0.2 aM to 20 fM were used to determine the limit of detection. HSV‐2, HPV‐16, HPV‐18, and HPV‐31 dsDNA templates were included to evaluate assay specificity.

### 4.9 Clinical Sample Testing and Validation

HSV‐1 clinical swab samples were processed using the DNeasy Blood and Tissue Kit according to the manufacturer's instructions. Briefly, 5 µL of the extracted HSV‐1 DNA was subjected to RPA amplification at 37°C for 10 min, followed by transfer of 1 µL of the RPA product into the CRISPR–Cas12a assay containing phosphate‐modified ssDNA reporters.

To validate the assay results, real‐time qPCR was performed on the extracted clinical samples using the GoTaq qPCR Kit. The 10 µL qPCR reaction mixture consisted of 5 µL 2× GoTaq qPCR Master Mix, 1.5 µL 20× primer/probe mixture (containing 10 µm forward primer, 10 µm reverse primer, and 5 µm hydrolysis probe), 1 µL DNA extract, and 2.5 µL nuclease‐free water. Thermal cycling conditions included an initial activation step at 95°C for 2 min, followed by 40 cycles of denaturation at 95°C for 15 s and annealing/extension at 60°C for 1 min. Fluorescence signals were recorded during each cycle using a CFX96 Touch Real‐Time PCR Detection System (Bio‐Rad, CA, USA; RRID: SCR_018064).

To evaluate the diagnostic performance, real‐time qPCR was used as the reference method. Based on the concordance between the Phos‐CRISPR and qPCR results, samples were classified as true positive (TP), true negative (TN), false positive (FP), or false negative (FN). According to previously reported methods [38], sensitivity, specificity, and accuracy were calculated using the following equations:

$$\mathrm{Sensitivity}:\frac{TP}{TP+FN}\times 100\%$$

$$\mathrm{Specificity}:\frac{TN}{TN+FP}\times 100\%$$

$$\mathrm{Accuracy}:\frac{TP+TN}{TP+TN+FP+FN}\times 100\%$$

## Author Contributions

**Rui Yang**: conceptualization, methodology, data curation, investigation, validation, formal analysis, visualization, Writing – original draft, Writing – review and editing. **Chong Guo**: methodology, data curation, investigation, visualization, writing – review and editing. **Minjie Pei**: methodology, investigation, validation, writing – review and editing. **Jiongyu Zhang**: investigation, validation, writing – review and editing, visualization. **Xin Guan**: investigation, validation, visualization, writing – review and editing. **Shuo Zhang**: validation, visualization, writing – review and editing. **Xingye Liu**: investigation, writing – review and editing. **Kurt T. Schalper**: investigation, writing – review and editing. **Lori Avery**: resources, writing – review and editing. **Rocco Lasala**: writing – review and editing, resources. **David Banach**: writing – review and editing, resources. **Changchun Liu**: conceptualization, investigation, writing – review and editing, resources, project administration, funding acquisition, supervision.

## Conflicts of Interest

The authors declare no conflicts of interest.

## Supporting information




**Supporting File**: advs78445‐sup‐0001‐SuppMat.pdf

## Data Availability

The data that support the findings of this study are available from the corresponding author upon reasonable request.
