# Supplementary material for: Phosphate‐Engineered DNA Reporters Boost CRISPR‐Cas12a Trans‐Cleavage for Rapid and Sensitive Nucleic Acid Detection
Source: Adv Sci (Weinh). 2026 Oct 25:e78445. Online ahead of print. doi: 10.1002/advs.78445 (PMC13696524; doi:10.1002/advs.78445)
Supplement: Supplementary file 1 [file ADVS-9999-e78445-s001.pdf]

Supporting Information

Phosphate-Engineered DNA Reporters Boost CRISPR-Cas12a *Trans*-Cleavage for Rapid and Sensitive Nucleic Acid Detection

Rui Yang[1,2], Chong Guo[1,2], Minjie Pei[1,2], Jiongyu Zhang[1], Xin Guan[1,2], Shuo Zhang[1,2], Xingye Liu[1,2], Kurt T. Schalper[1,2], Lori Avery[3], Rocco LaSala[3], David Banach[4], and Changchun Liu[1]*

1. *Department of Biomedical Engineering, University of Connecticut Health Center, Farmington, Connecticut 06030, United States*
2. *Department of Biomedical Engineering, University of Connecticut, Storrs, Connecticut 06269, United States*
3. *Department of Pathology and Laboratory Medicine, University of Connecticut Health Center, Farmington, CT, 06030, USA*
4. *Department of Medicine, Division of Infectious Diseases, University of Connecticut Health Center, Farmington, CT, 06030, USA*

*Corresponding author

Dr. Changchun Liu

Department of Biomedical Engineering

University of Connecticut Health Center

263 Farmington Avenue

Farmington, CT 06030

Phone: (860) 679-2565

E-mail: chaliu@uchc.edu

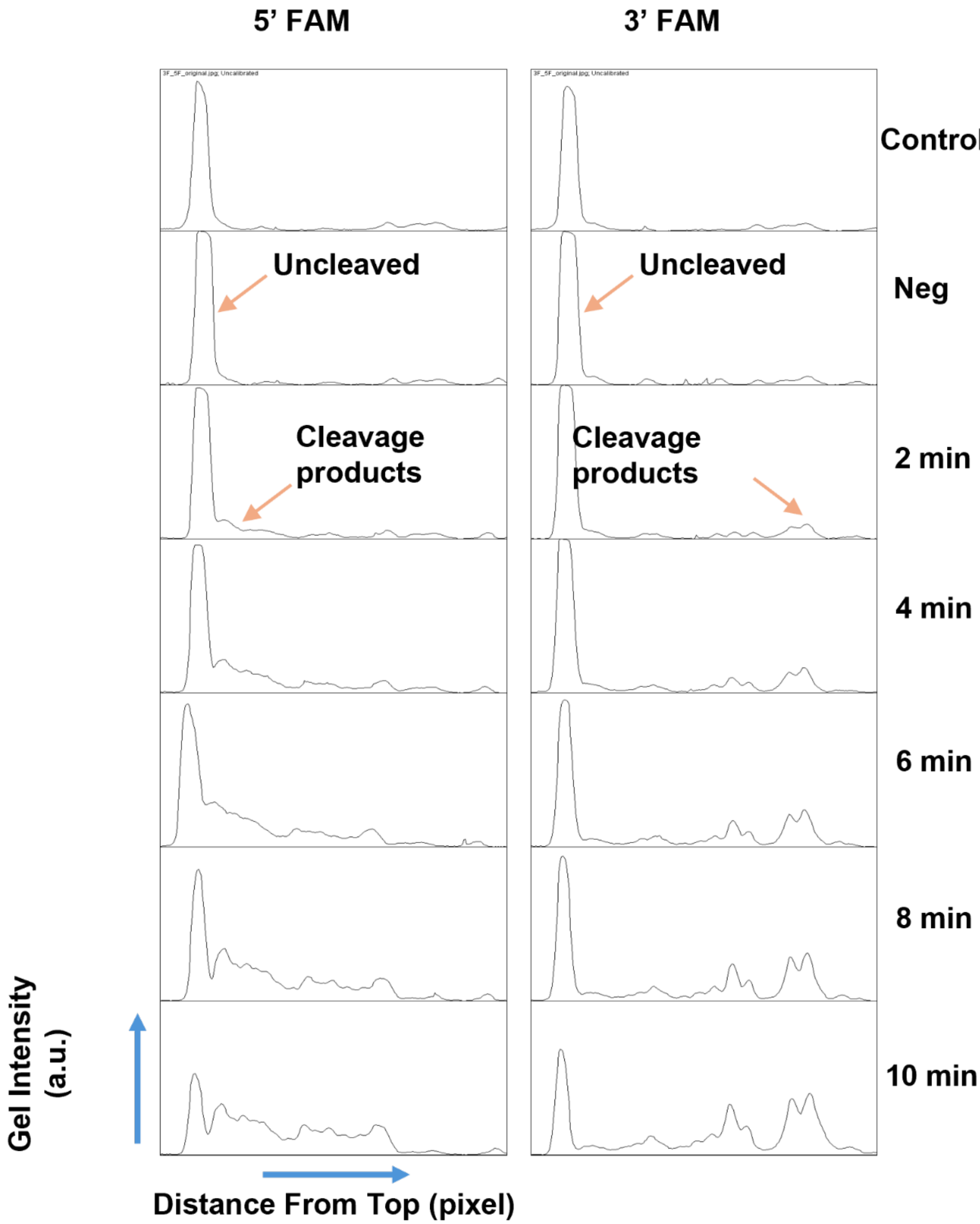

Figure S1. Intensity plots of each lane generated using ImageJ from the background-subtracted gels shown in **Fig. 2b**.

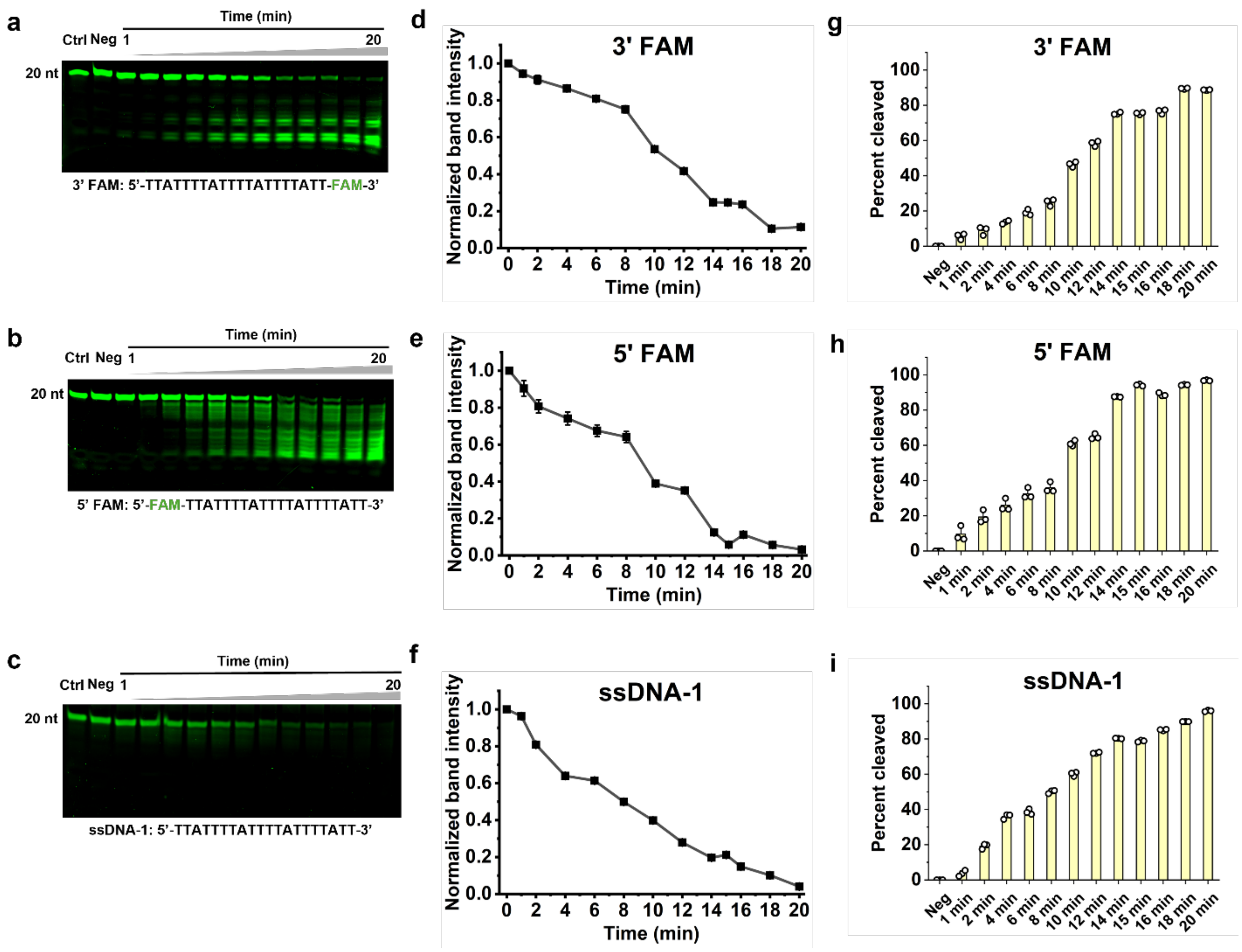

Figure S2. a–c, Denaturing gels showing the *trans*-cleavage of 3′-FAM-labeled, 5′-FAM-labeled, and unlabeled ssDNA substrates. Control lanes contained only ssDNA substrate to indicate the full-length 20-nt length band. **d–f,** Comparison of normalized band intensities for 3′-FAM-labeled, 5′-FAM-labeled, and unlabeled ssDNA substrates. **g–i,** Comparison of percent cleavage for 3′-FAM-labeled, 5′-FAM-labeled, and unlabeled ssDNA substrates. Data are shown as mean ± s.d. (n = 3).

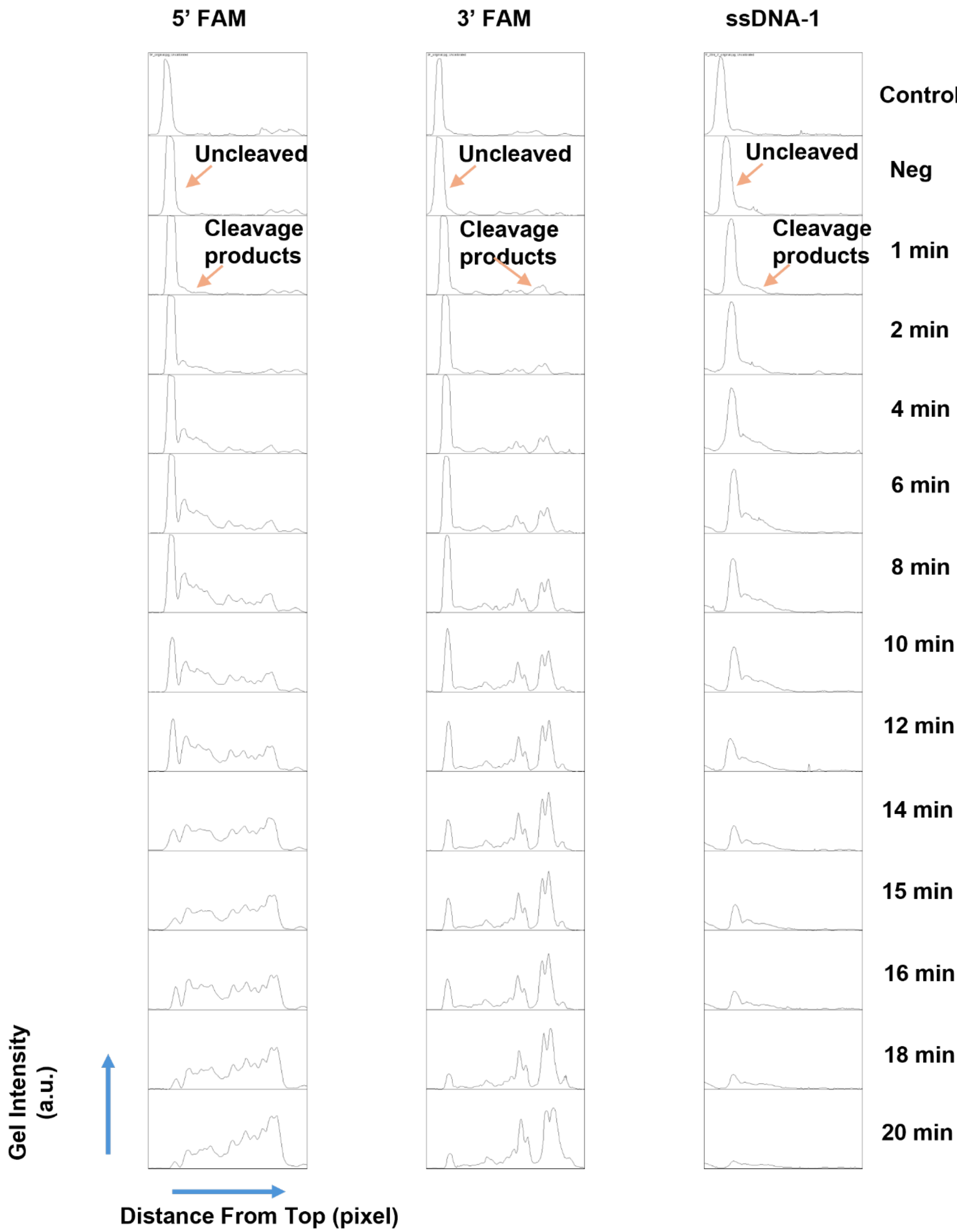

Figure S3. Intensity plots of each lane generated using ImageJ from the background-subtracted gels shown in **Fig. S2a–c**.

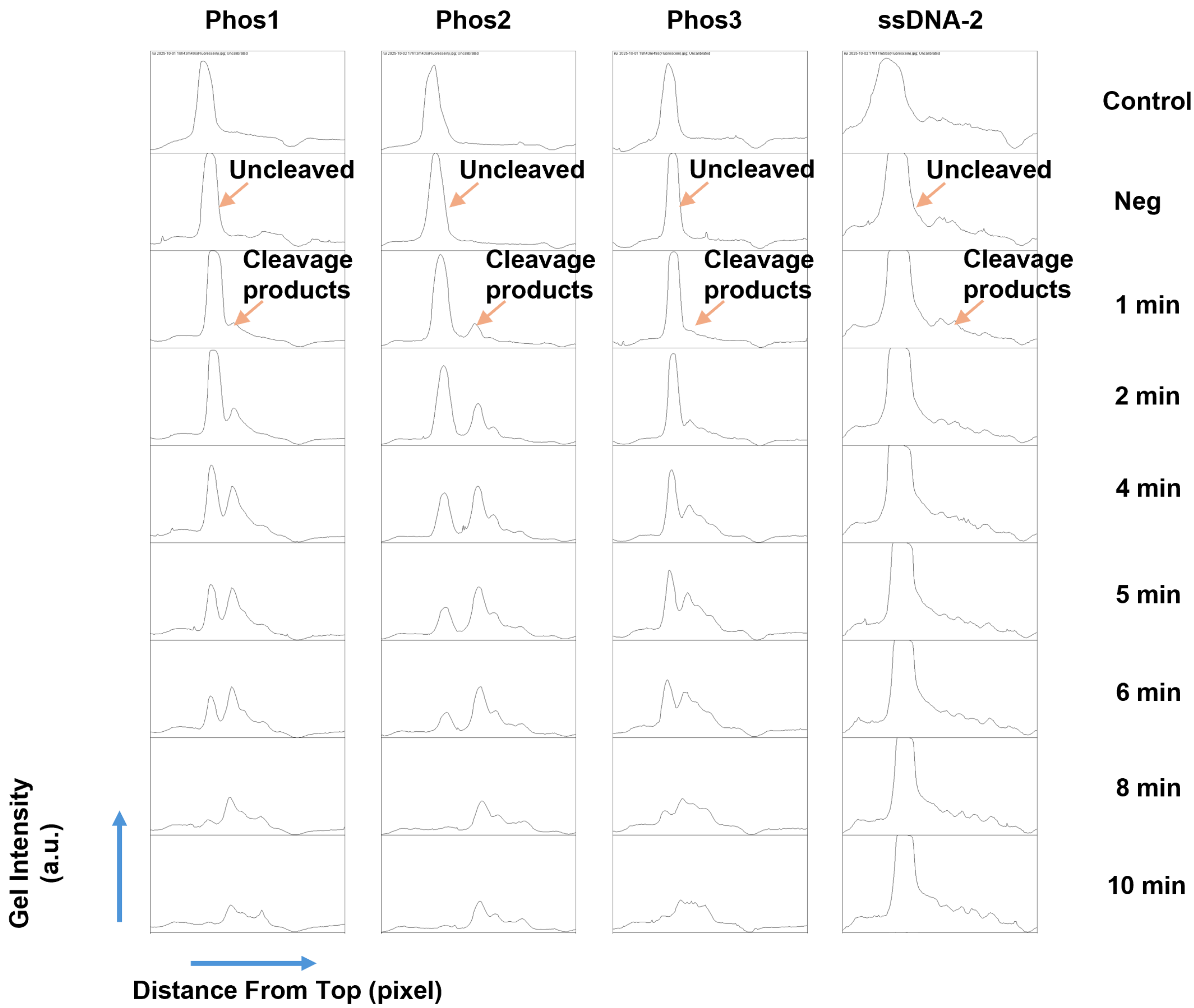

Figure S4. Intensity plots of each lane generated using ImageJ from the background-subtracted gels shown in **Fig. 3b**.

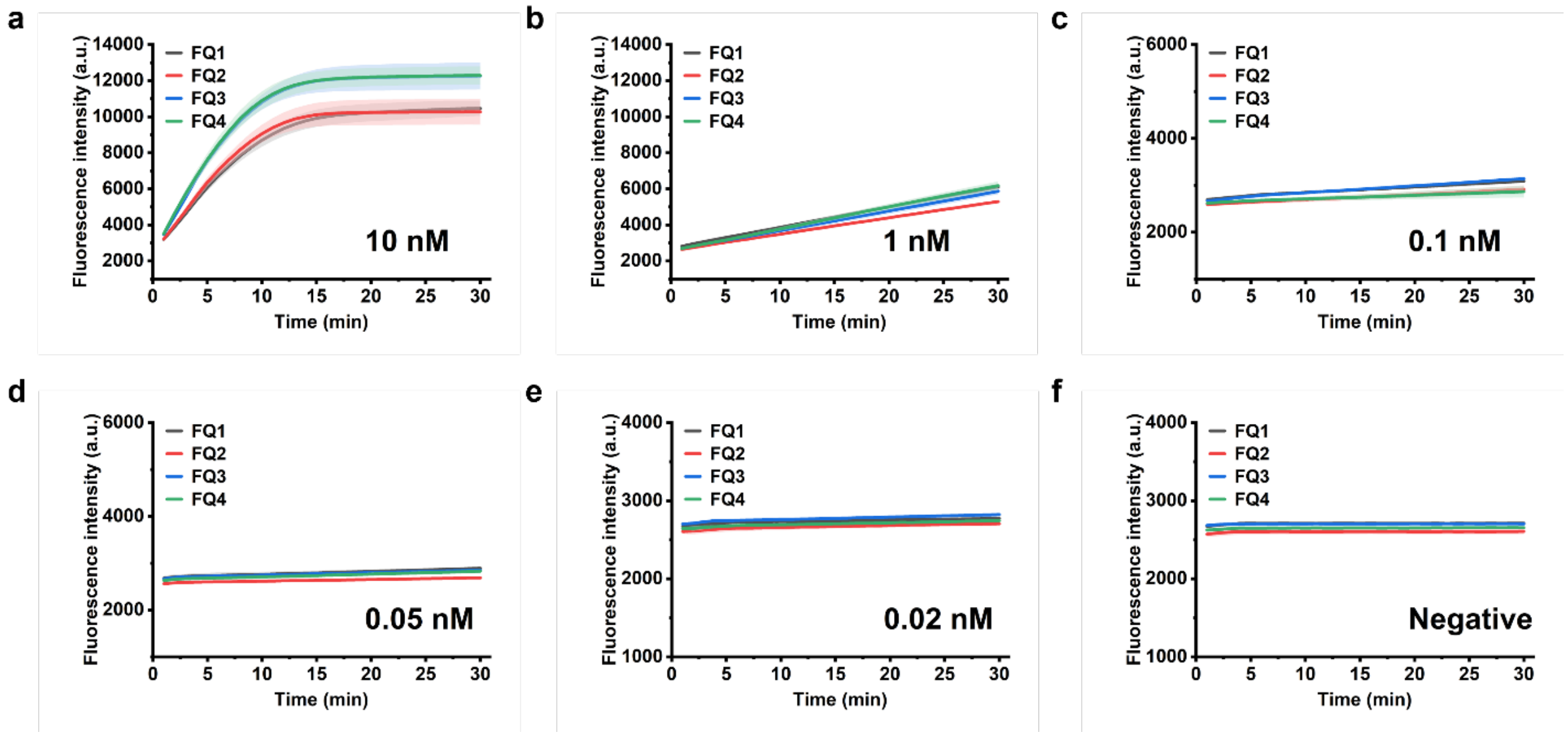

Figure S5. a–f, Comparison of fluorescence kinetics for three phosphate-modified ssDNA FQ reporters (FQ1–FQ3) and one unmodified ssDNA FQ reporter (FQ4) at different target concentrations.

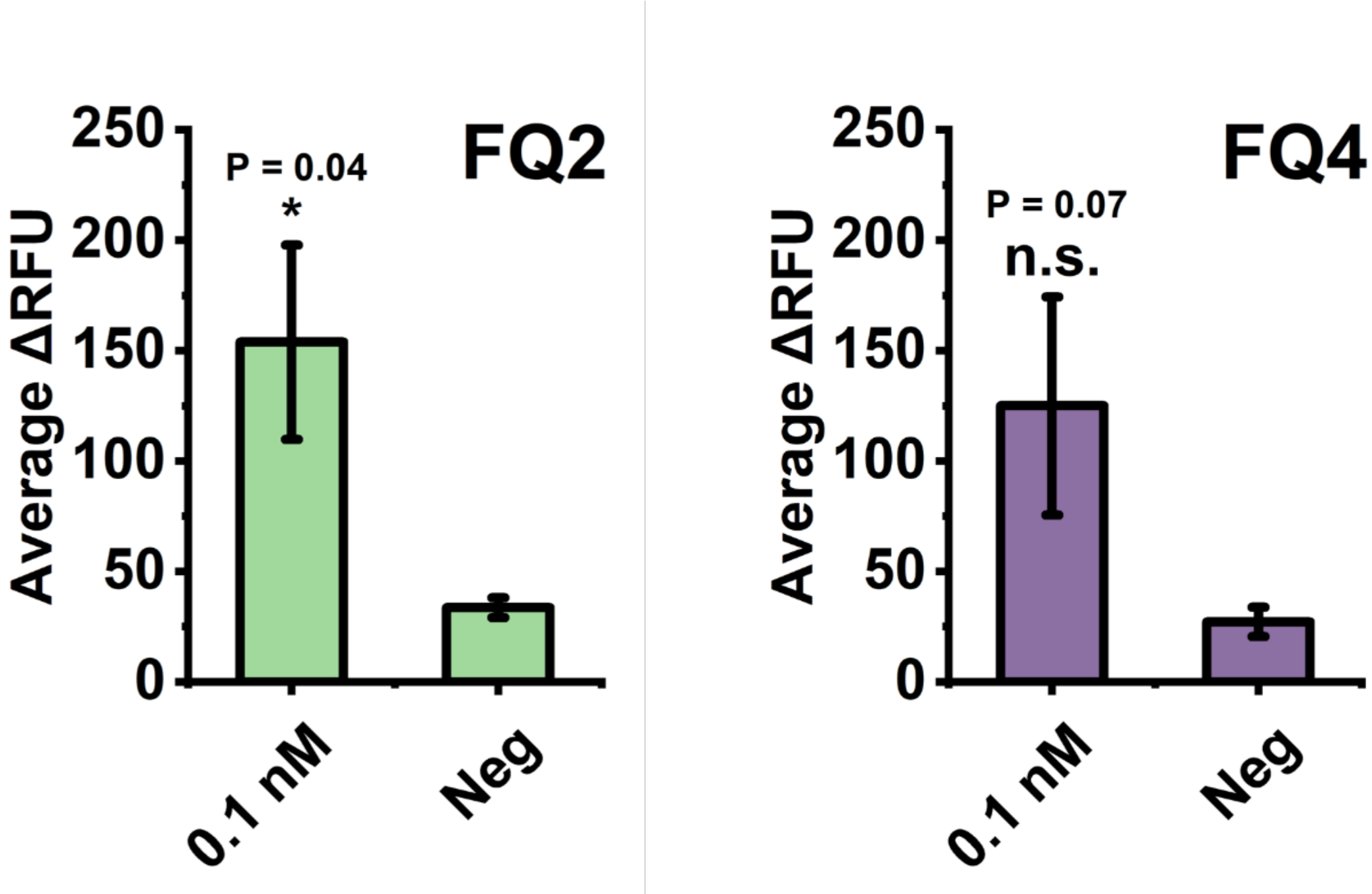

Figure S6. Comparison of endpoint fluorescence increase in CRISPR reactions using the 5′-phosphorylated FQ reporter (FQ2) and unmodified ssDNA FQ reporter (FQ4) at 15 min.

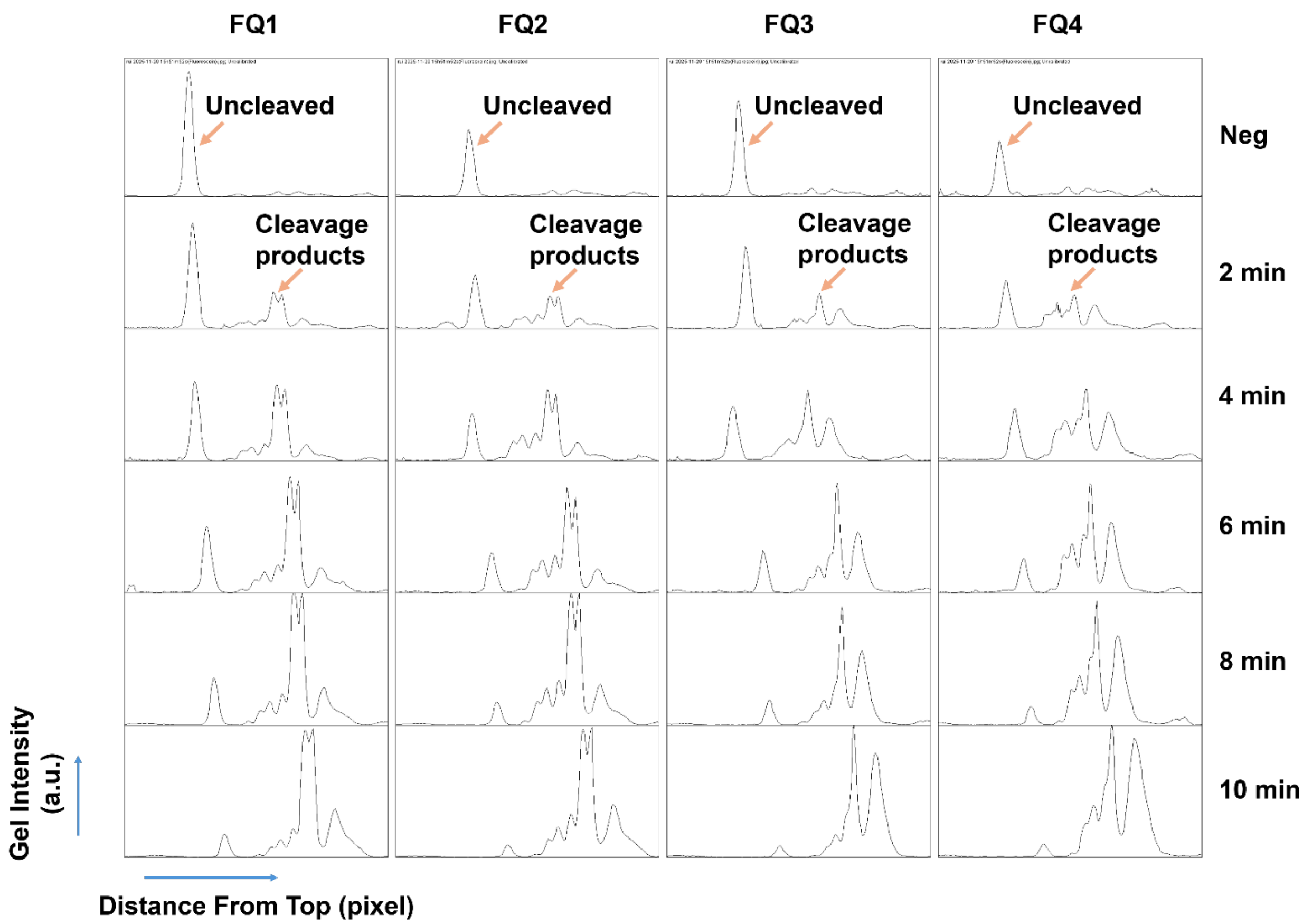

Figure S7. Intensity plots of each lane generated using ImageJ from the background-subtracted gels shown in **Fig. 4d**.

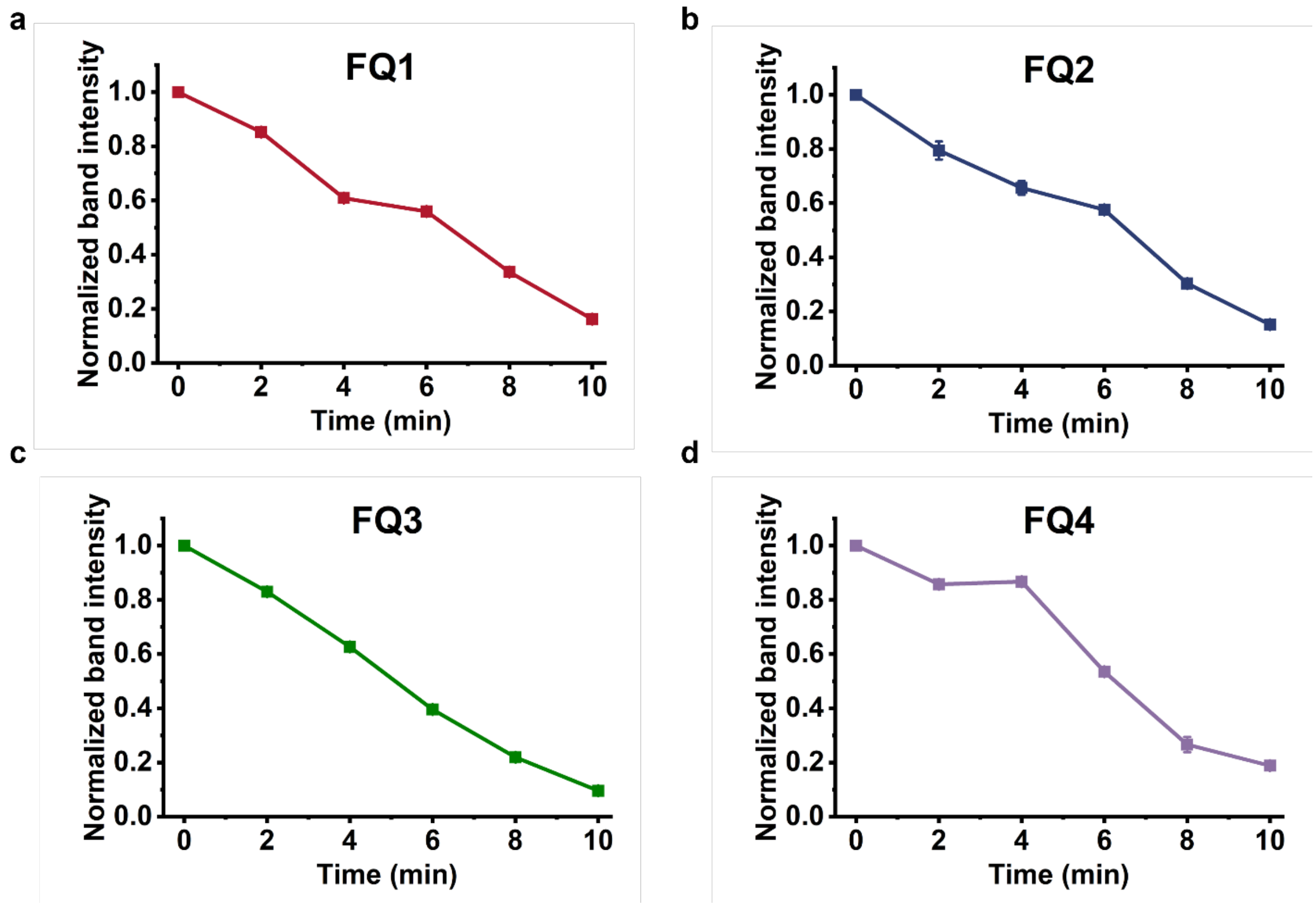

Figure S8. a–d, Comparison of normalized band intensities for phosphate-modified ssDNA FQ reporters (FQ1–FQ3) and the unmodified ssDNA FQ reporter (FQ4). Data are shown as mean ± s.d. (n = 3).

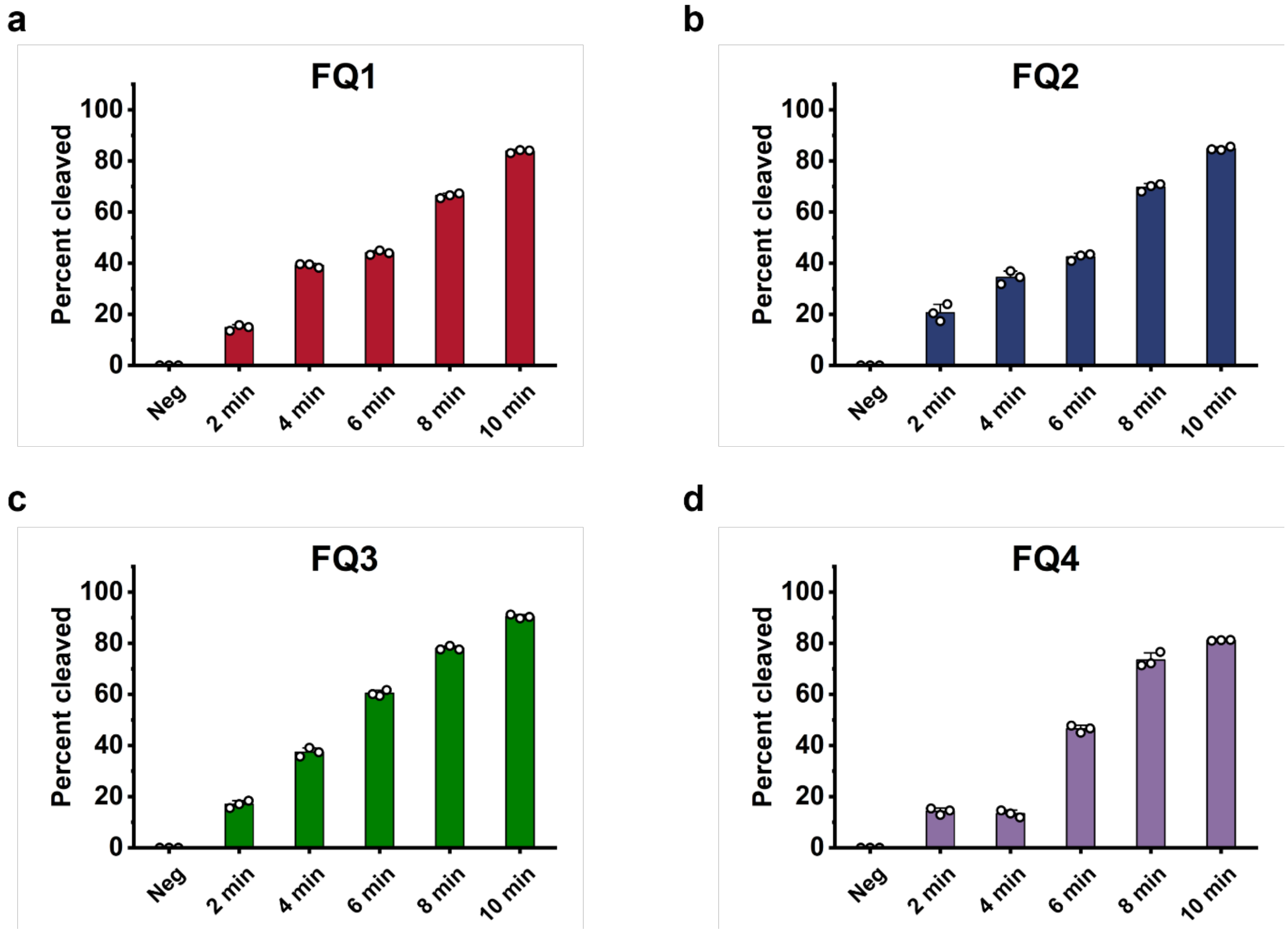

Figure S9. a–d, Comparison of percent cleavage for phosphate-modified ssDNA FQ reporters (FQ1–FQ3) and the unmodified ssDNA FQ reporter (FQ4). Data are shown as mean ± s.d. (n = 3).

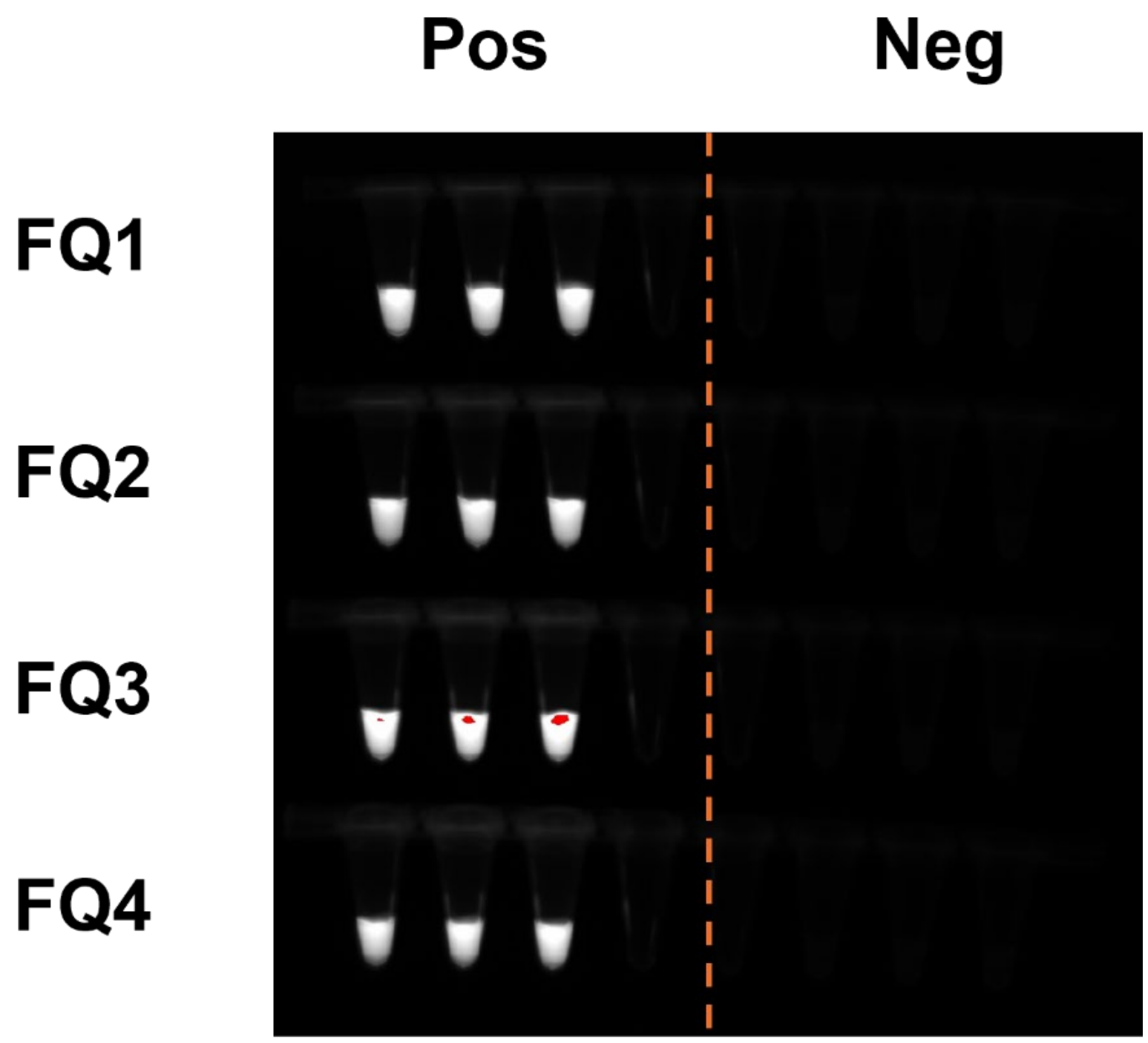

Figure S10. Endpoint fluorescence image under UV light of DNase I digestion on phosphate-modified ssDNA FQ reporters (FQ1–FQ3) and the unmodified ssDNA FQ reporter. Red highlights represent strong fluorescence intensity.

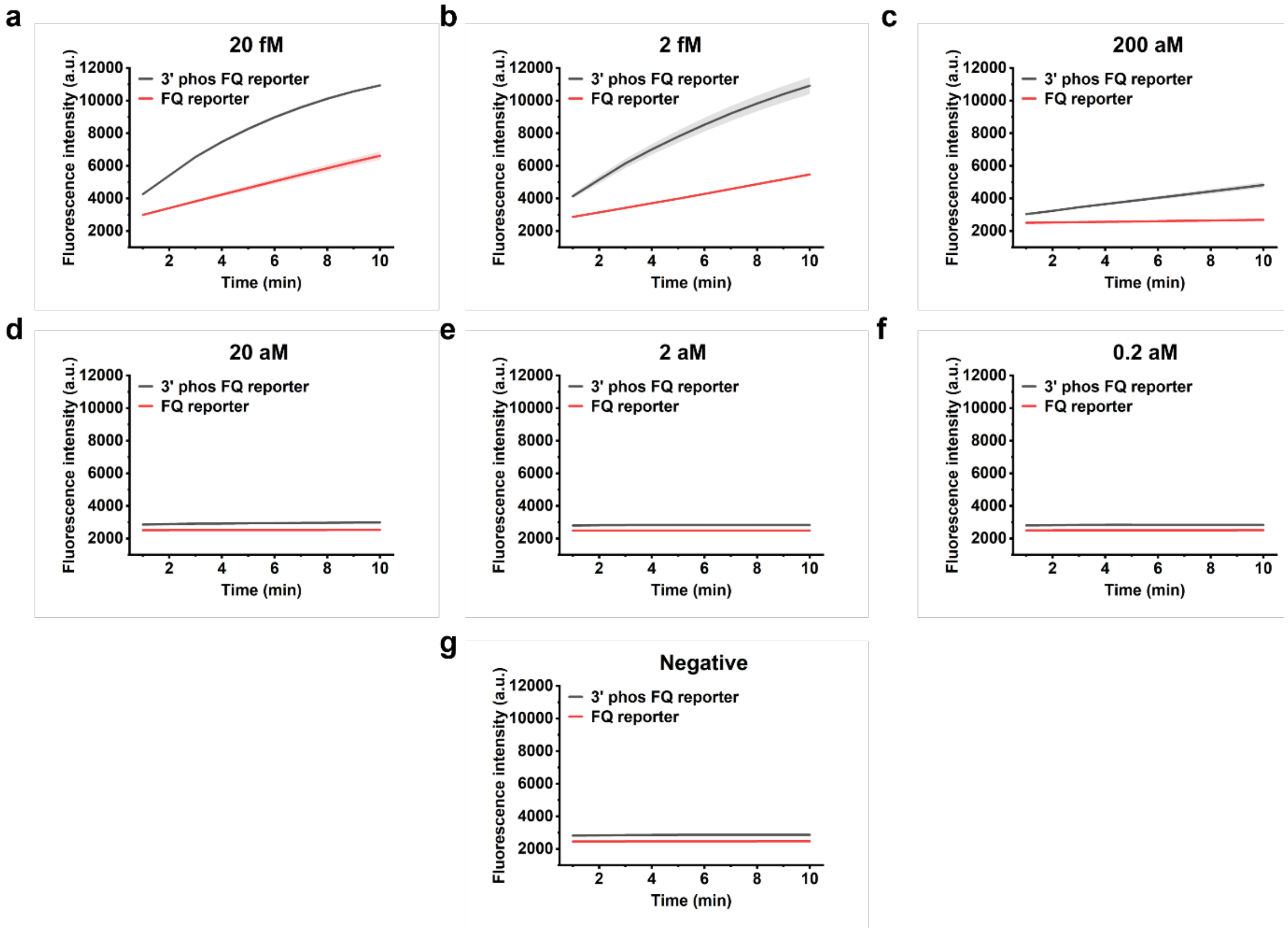

Figure S11. a–g, Comparison of fluorescence kinetics between Phos-CRISPR and conventional CRISPR assays using the 3′-phosphate FQ reporter and the unmodified FQ reporter, respectively.

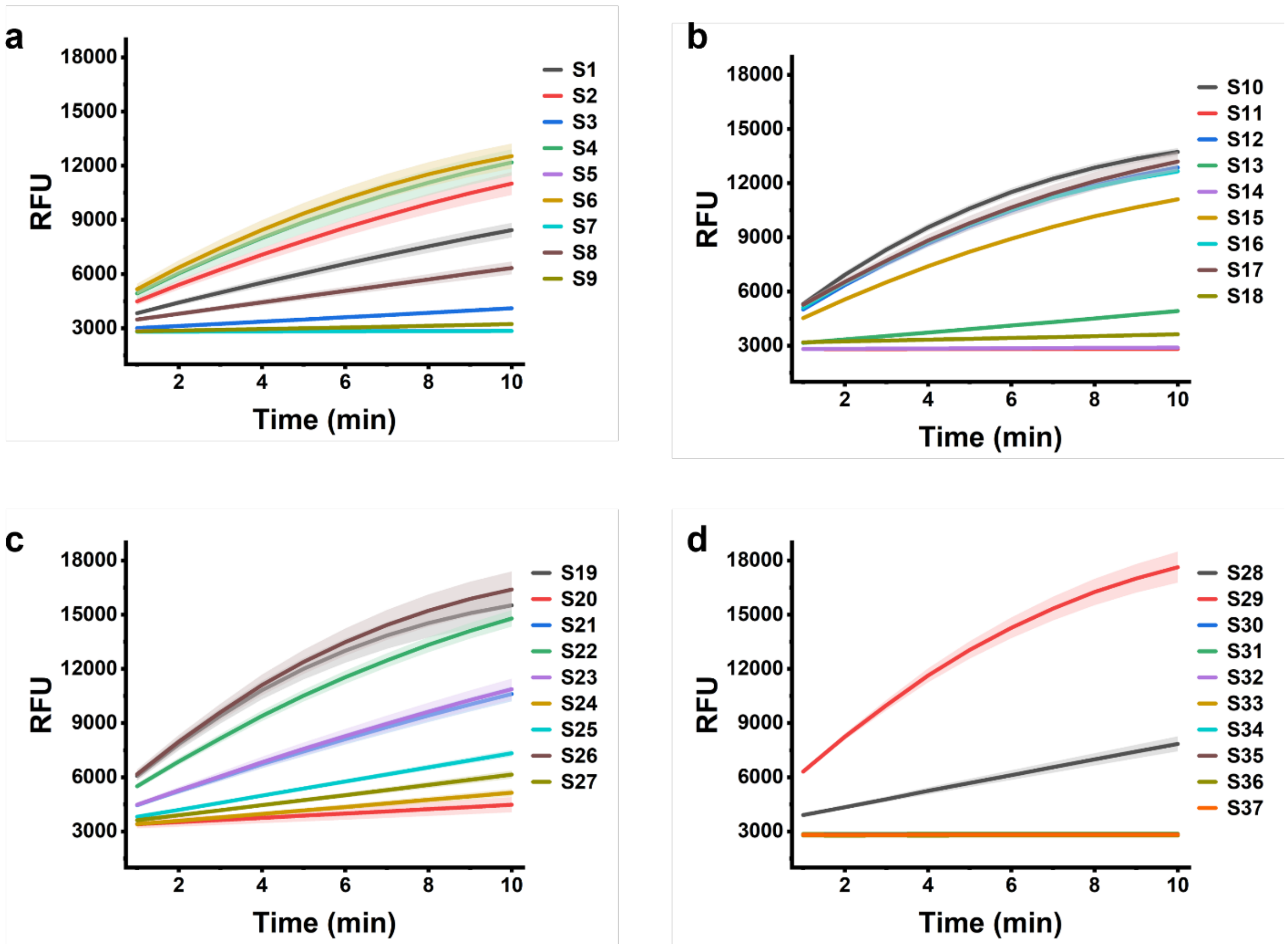

Figure S12. a–d, Fluorescence kinetics of the Phos-CRISPR assay with 3'phos FQ reporter for HSV-1 detection in 37 clinical swab samples.

Reporter	K_{cat} (s^{-1})	K_M (M)	K_{cat} / K_M ($M^{-1}s^{-1}$)	Reference
FQ reporter	0.025	1.24×10^{-6}	1.97×10^4	This work
3' phos FQ reporter	0.084	3.71×10^{-6}	2.26×10^4	This work
FQ reporter	0.09	2.13×10^{-7}	4.2×10^5	Zhang J. et al.[1]
FQ reporter	0.07	2.74×10^{-7}	2.5×10^5	Zhang J. et al.[1]
FQ reporter	17	1.01×10^{-6}	1.7×10^7	Chen J. et al.[2, 3]
FQ reporter	0.001	9×10^{-7}	1.7×10^3	Wörle E. et al. [4]
FQ reporter	0.00099	6.3×10^{-8}	1.57×10^4	Zhang W. et al. [5]

Table S1. Comparison of enzyme kinetic parameters obtained with the Phos-CRISPR assay and those reported in previous studies.

Sample	Phos-CRISPR with 3'phos FQ	qPCR	C_t value
S1	+++	+	29.70
S2	+++	+	27.67
S3	+++	+	32.35
S4	+++	+	27.59
S5	- - -	-	N/A
S6	+++	+	27.19
S7	- - -	-	N/A
S8	+++	+	30.85
S9	+++	+	34.32
S10	+++	+	24.95
S11	- - -	-	N/A
S12	+++	+	26.59
S13	+++	+	31.70
S14	- - -	+	39.50
S15	+++	+	26.98
S16	+++	+	25.45
S17	+++	+	25.80
S18	+++	+	34.16
S19	+++	+	22.48
S20	+++	+	33.72
S21	+++	+	28.67
S22	+++	+	24.98
S23	+++	+	29.00
S24	+++	+	32.83
S25	+++	+	30.14
S26	+++	+	23.77
S27	+++	+	31.89
S28	+++	+	30.97
S29	+++	+	21.99
S30	- - -	-	N/A
S31	- - -	-	N/A
S32	- - -	-	N/A
S33	- - -	-	N/A
S34	- - -	-	N/A
S35	- - -	-	N/A
S36	- - -	-	N/A
S37	- - -	-	N/A

Table S2. Comparison of the Phos-CRISPR assay using the 3'phos FQ reporter with qPCR for detection of HSV-1 DNA in 37 clinical swab samples. Each sample was tested three times by the Phos-CRISPR assay and once by qPCR. N/A represents Ct value ≥ 40.

Name	Sequence
5’ FAM	5’ FAM-TTATTTTATTTTATTTTATT
3’ FAM	TTATTTTATTTTATTTTATT-3’ FAM
Phos1	5’ Phos-TTGCCTACGCCATCAG-Phos 3’
Phos2	5’ Phos-TTGCCTACGCCATCAG
Phos3	TTGCCTACGCCATCAG-Phos 3’
ssDNA-1	TTATTTTATTTTATTTTATT
ssDNA-2	TTGCCTACGCCATCAG
FQ1	5’ Phos-FAM-TTATTTTATT-BHQ1-Phos 3’
FQ2	5’ Phos-FAM-TTATTTTATT-BHQ1
FQ3	5’ FAM-TTATTTTATT-BHQ1-Phos 3’
FQ4	5’ FAM-TTATTTTATT-BHQ1- 3’
FQ5nt	5’ FAM-TTATT-BHQ1- 3’
Primer F-RPA	AGCGCCCTCTGGATGCCAGACCTGTTTTTCAA
Primer R-RPA	GCGGTTCACCGCGTCTTTGAACATGACGAAGG
crRNA-T1	UAAUUUCUACUAAGUGUAGAUCGUCGCCGUCCAGCUCGACC
crRNA-HSV1	UAAUUUCUACUAAGUGUAGAUACGGGGAGGAGUUCGAGAAG
Primer F-qPCR	ATGCCAGACCTGTTTTTCAA
Primer R-qPCR	GTCTTTGAACATGACGAAGG
Probe-qPCR	/56-FAM/CAGCTCCTG/ZEN/GATGGGTATCTGCTC/3IABkFQ/
T1 DNA target	GCCGGGGTGGTGCCCATCCTGGTCGAGCTGGACGGCGACGTAA ACGGCCACAAGC
HSV 1 dsDNA	AGCGCCCTCTGGATGCCAGACCTGTTTTTCAAGCGCCTGATTCG CCACCTGGACGGCGAGAAGAACGTCACATGGACCCTGTTCGAC CGGGACACCAGCATGTCGCTCGCCGACTTTCACGGGGAGGAGT TCGAGAAGCTCTACCAGCACCTCGAGGTCATGGGGTTCGGCGA GCAGATACCCATCCAGGAGCTGGCCTATGGCATTGTGCGCAGT GCGGCCACGACCGGGAGCCCCTTCGTCATGTTCAAAGACGCGG TGAACCGC

Table S3. Related nucleic acid sequences.

References

1. Zhang, J., Z. Li, C. Guo, et al., Intrinsic RNA Targeting Triggers Indiscriminate DNase Activity of CRISPR-Cas12a. *Angew Chem Int Ed Engl*, 2024. **63**(20): p. e202403123. https://doi.org/10.1002/anie.202403123
2. Chen, J.S., E. Ma, L.B. Harrington, et al., CRISPR-Cas12a target binding unleashes indiscriminate single-stranded DNase activity. *Science*, 2018. **360**(6387): p. 436-439. https://doi.org/10.1126/science.aar6245
3. Erratum for the Report "CRISPR-Cas12a target binding unleashes indiscriminate single-stranded DNase activity" by J. S. Chen, E. Ma, L. B. Harrington, M. Da Costa, X. Tian, J. M. Palefsky, J. A. Doudna. *Science*, 2021. **371**(6531). https://doi.org/10.1126/science.abh0317
